# Defects in meiosis I contribute to the genesis of androgenetic hydatidiform moles

**DOI:** 10.1172/JCI170669

**Published:** 2024-11-15

**Authors:** Maryam Rezaei, Manqi Liang, Zeynep Yalcin, Jacinta H. Martin, Parinaz Kazemi, Eric Bareke, Zhao-Jia Ge, Majid Fardaei, Claudio Benadiva, Reda Hemida, Adnan Hassan, Geoffrey J. Maher, Ebtesam Abdalla, William Buckett, Pierre-Adrien Bolze, Iqbaljit Sandhu, Onur Duman, Suraksha Agrawal, JianHua Qian, Jalal Vallian Broojeni, Lavi Bhati, Pierre Miron, Fabienne Allias, Amal Selim, Rosemary A. Fisher, Michael J. Seckl, Philippe Sauthier, Isabelle Touitou, Seang Lin Tan, Jacek Majewski, Teruko Taketo, Rima Slim

**Affiliations:** 1 Department of Human Genetics, McGill University Health Centre, Montreal, Quebec, Canada.; 2 Department of Biology, McGill University, Montreal, Quebec, Canada.; 3 Department of Medical Genetics, Shiraz University of Medical Sciences, Shiraz, Iran.; 4 Center for Advanced Reproductive Services, Farmington, Connecticut, USA.; 5 Department of Obstetrics and Gynecology, Mansoura University, Mansoura, Egypt.; 6 Department of Obstetrics and Gynecology, Jordan Hospital, Amman, Jordan.; 7 Department of Surgery and Cancer, Imperial College London, London, United Kingdom.; 8 Department of Human Genetics, Medical Research Institute, Alexandria University, Alexandria, Egypt.; 9 Department of Obstetrics and Gynecology, McGill University Health Centre, Montreal, Quebec, Canada.; 10 Université Lyon 1, Service de Chirurgie Gynécologique et Ontologique, Obstétrique, Centre Français de Référence des Maladies Trophoblastiques, Hospices Civils de Lyon, Hôpital Lyon Sud, Pierre Bénite, France.; 11 Security Research Center, Concordia University, Montreal, Quebec, Canada.; 12 Department of Medical Genetics, Sanjay Gandhi Postgraduate Institute of Medical Sciences, Lucknow, India.; 13 Department of Gynecology, The First Affiliated Hospital, Zhejiang University School of Medicine, Hangzhou, China.; 14 Centre d’Aide Médicale à la Procréation Fertilys, Laval, Quebec, Canada.; 15 Institut National de Recherche Scientifique–Centre Armand-Frappier Santé Biotechnologie, Laval, Quebec, Canada.; 16 Department of Pathology, Hospices Civils de Lyon, Centre, Hospitalier Lyon Sud, Pierre-Bénite, France.; 17 Department of Medical Biochemistry and Molecular Biology, Mansoura University, Mansoura, Egypt.; 18 Department of Obstetrics and Gynecology, Gynecologic Oncology Division, Centre Hospitalier de l’Université de Montréal, Réseau des Maladies Trophoblastiques du Québec, Montreal, Quebec, Canada.; 19 Department of Genetics CHU of Montpellier, University of Montpellier, INSERM, Montpellier, France.; 20 OriginElle Fertility Clinic and Women’s Health Centre, Montreal, Quebec, Canada.; 21 Department of Surgery, McGill University Health Centre, Montreal, Quebec, Canada.

**Keywords:** Genetics, Reproductive biology, Fertility, Monogenic diseases

## Abstract

To identify novel genes responsible for recurrent hydatidiform moles (HMs), we performed exome sequencing on 75 unrelated patients who were negative for mutations in the known genes. We identified biallelic deleterious variants in 6 genes, *FOXL2*, *MAJIN*, *KASH5*, *SYCP2*, *MEIOB*, and *HFM1*, in patients with androgenetic HMs, including a familial case of 3 affected members. Five of these genes are essential for meiosis I, and their deficiencies lead to premature ovarian insufficiency. Advanced maternal age is the strongest risk factor for sporadic androgenetic HM, which affects 1 in every 600 pregnancies. We studied *Hfm1^–/–^* female mice and found that these mice lost all their oocytes before puberty but retained some at younger ages. Oocytes from *Hfm1^–/–^* mice initiated meiotic maturation and extruded the first polar bodies in culture; however, their meiotic spindles were often positioned parallel, instead of perpendicular, to the ooplasmic membrane at telophase I, and some oocytes extruded the entire spindle with all the chromosomes into the polar bodies at metaphase II, a mechanism we previously reported in *Mei1^–/–^* oocytes. The occurrence of a common mechanism in two mouse models argues in favor of its plausibility at the origin of androgenetic HM formation in humans.

## Abstract 2

We identified pathogenic variants in six meiotic genes in patients with recurrent androgenetic moles and modeled the loss of the mechanism of androgenesis in *Hfm1*-null mice.

## Graphical abstract

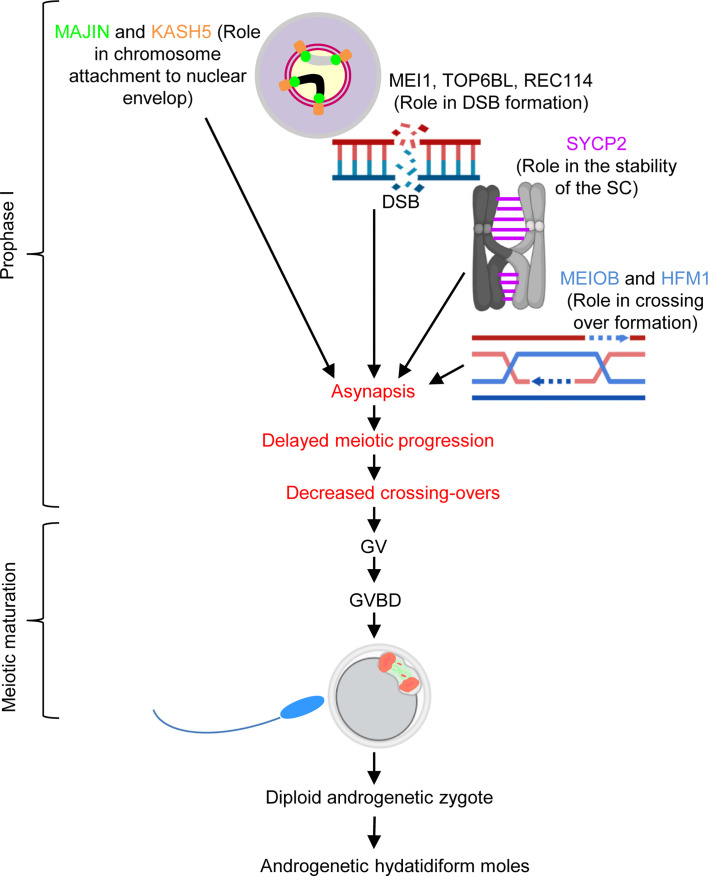

## Introduction

Hydatidiform moles (HMs) are abnormal human pregnancies with impaired embryonic development and excessive trophoblastic proliferation. Common HMs are sporadic and non-recurrent and affect 1 in every 600 pregnancies in Western countries (1). Based on morphological evaluation, HMs are subdivided into complete (CHM) and partial (PHM). Common sporadic CHMs have diploid androgenetic genome with all the chromosomes being inherited from the father and originate from the fertilization of an oocyte by 1 spermatozoon (monospermic) in 85%–90% of cases or 2 spermatozoa (dispermic) in 10%–15% of cases (2, 3). Common sporadic PHMs are mostly triploid and derive from the fertilization of an oocyte by 2 spermatozoids in 99% of cases (3).

Recurrent HMs (RHMs) are defined by the occurrence of at least 2 HMs in the same patient, and some of them are familial, occurring in at least 2 family members. To date, recessive mutations in 4 genes coding for members of the subcortical maternal complex (SCMC), a structural protein complex that is uniquely present in mammalian oocytes, have been found to be responsible for RHM. *NLRP7* is the major gene for RHM and explains the genetic etiology of 55% of patients. *KHDC3L* explains the genetic etiology of 5% of patients. *PADI6* and *NLRP5* explain the genetic etiology of 1% and 0.5% of cases, respectively (4). The SCMC is required in the oocyte for RNA storage, ribosome and organelle distribution, and the activation of zygotic genome transcription (5). Biallelic mutations in *NLRP7* and *KHDC3L* in patients are associated with highly recurrent HMs with diploid biparental genomes. In these tissues, the molar phenotype is believed to be caused by the altered structure and function of the SCMC, and is associated with decreased de novo DNA methylation in oocytes, impaired embryonic tissue differentiation, and increased trophoblastic proliferation (reviewed in ref. 4). Biallelic mutations in each of *MEI1*, *TOP6BL*, and *REC114* explain the genetic etiology of 0.5% of patients with RHM (4). Collectively, these 3 genes have roles in double-strand break (DSB) formation in early meiosis I, and their biallelic mutations are associated with androgenetic monospermic RHM (6) as well as primary or secondary female infertility, early embryonic arrest during the preimplantation period, recurrent miscarriage (MC), and male infertility (7–13). Altogether, the known genes explain the etiology of 63% of patients with RHM, and the remaining patients with unexplained etiology are highly heterogeneous (14).

In this study, we performed whole exome sequencing (WES) on 75 probands with RHM, who were negative for recessive mutations in *NLRP7* and *KHDC3L*, to identify novel causative genes for RHM pathogenesis. We found biallelic deleterious variants in 6 novel genes in patients with recurrent androgenetic CHM (AnCHM) and modeled the mechanism of AnCHM in *Hfm1^–/–^* female mice.

## Results

### Identification of deleterious variants in FOXL2, MAJIN, KASH5, SYCP2, HFM1, and MEIOB.

WES on a total of 75 unrelated patients with at least 2 HMs (including all histopathological and genotypic types), who were negative for recessive mutations in *NLRP7* and *KHDC3L*, revealed the presence of deleterious biallelic variants in 5 genes with known roles in meiosis I in 5 unrelated probands.

**Figure 1:**
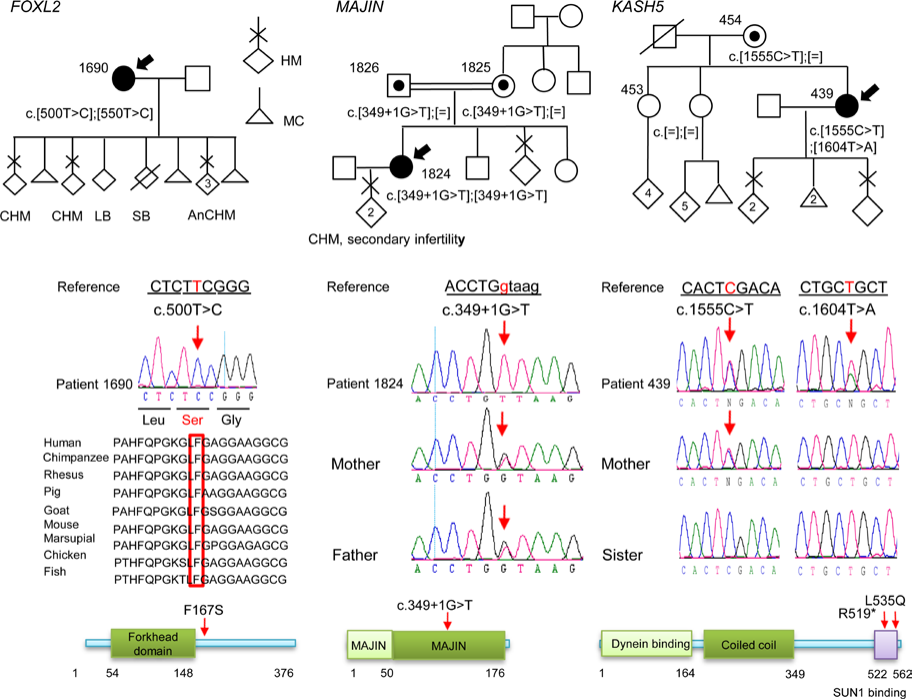
Pedigrees, Sanger sequencing, and segregation of the variants in *FOXL2*, *MAJIN*, and *KASH5*. The probands are indicated by arrows. Amino acid numbering is given below the protein structure. On the protein structure, the red arrows indicate the positions of the variants seen in a recessive state. HM, hydatidiform mole; CHM, complete HM; AnCHM, androgenetic CHM; LB, live birth; SB, stillbirth; MC, miscarriage.

In *FOXL2* (forkhead box L2), analysis of the exome of patient 1690 (South Asian), with 5 CHMs, 3 MCs, 1 stillbirth, and 1 live birth, identified a novel missense variant, NM_023067:c.500T>C, p.(Phe167Ser), in a homozygous state located in a run of homozygosity (ROH) of 10.5 Mb (Figure 1 and Supplemental Figure 1; supplemental material available online with this article; https://doi.org/10.1172/JCI170669DS1). This variant was classified as a variant of unknown significance (VUS) with 4 points (4 pathogenic and 0 benign) by the American College of Medical Genetics and Genomics (ACMG) guidelines (15) using VarSome (16) and was predicted to be deleterious by Combined Annotation Dependent Depletion (CADD) (24.5) (17), Polymorphism Phenotyping v2 (PolyPhen-2) (0.9) (18), and Sorting Intolerant from Tolerant (SIFT) (0.02); it is highly conserved from fish to humans (Figure 1). Repeating the WES (with 131× average coverage) on this patient did not identify any other candidate variant (Supplemental Figure 2).

In *MAJIN* (membrane-anchored junction protein), analysis of the exome of patient 1824 (Italian), with 2 HMs followed by secondary infertility and substantially reduced bilateral ovarian volumes (1.1 cm^3^ and 2.5 cm^3^ at the age of 25 years as compared with the normal volume, 6.1 cm^3^ in women under the age of 30 years), revealed a novel variant affecting the splice donor of exon 6, NM_001037225.3:c.349+1G>T, in a homozygous state and located in an ROH of 48.3 Mb (Figure 1 and Supplemental Figure 3). This variant is predicted to be likely pathogenic (LP) by the ACMG. The variant segregated from both parents, who are second- or third-degree cousins. Interestingly, the patient’s mother reported that she had a molar pregnancy at age 21 and that her menses had always been irregular.

In *KASH5* (KASH domain–containing protein 5), analysis of the exome of patient 439 (Jordanian), with 3 HMs and 2 MCs, identified 2 variants, NM_144688:c.1555C>T, p.(Arg519*), and c.1604T>A, p.(Leu535Gln), in a heterozygous state (Figure 1 and Supplemental Figure 4). Variant p.R519* has a CADD score of 36 and is predicted to be VUS with 1 point (1 pathogenic and 0 benign) by the ACMG. The second variant, L535Q, has a CADD score of 25.6 and is predicted to be pathogenic by ClinVar (19) and VUS with 2 points (2 pathogenic and 0 benign) by the ACMG. The L535Q variant was previously reported in a homozygous state by Fakhro et al. (20) in 2 infertile Qatari brothers with azoospermia. Sanger sequencing validated the 2 variants in the proband and showed that only one of them, p.R519*, was inherited from her mother, and therefore, the 2 variants are most likely on 2 different parental chromosomes.

**Figure 2:**
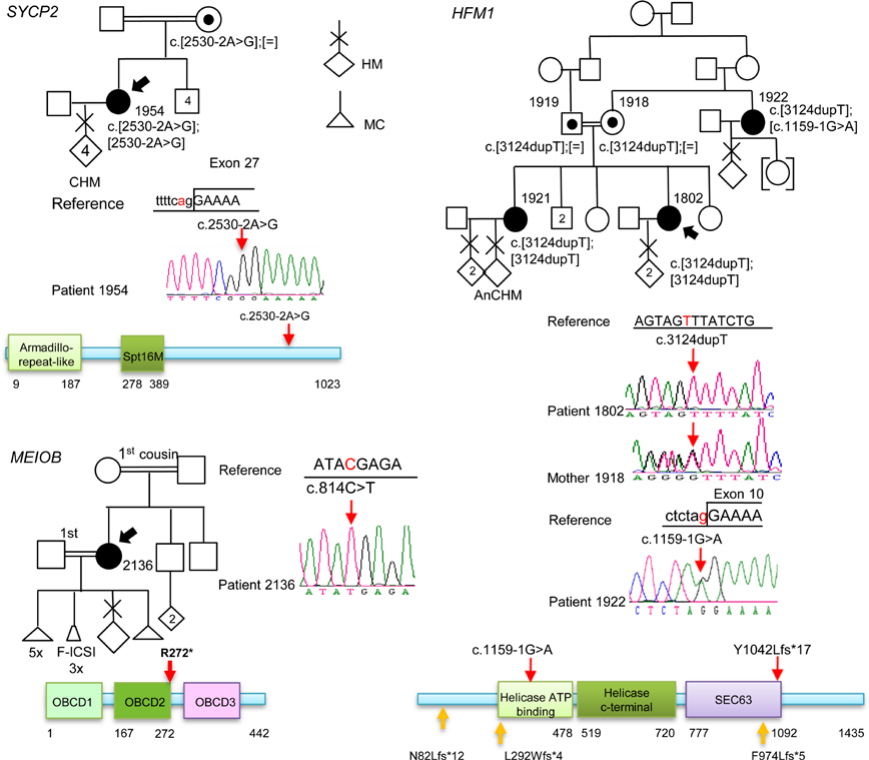
Pedigrees, Sanger sequencing, and segregation of the variants in *SYCP2*, *MEIOB*, and *HFM1*. The probands are indicated by arrows. Amino acid numbering is given below the protein structure. On the protein structure, the red arrows indicate variants seen in a recessive state, and the orange arrows indicate variants seen as a single heterozygous variant. HM, hydatidiform mole; CHM, complete HM; AnCHM, androgenetic CHM; F-ICSI, failed intracytoplasmic sperm injection.

In *SYCP2* (synaptonemal complex protein 2), analysis of the exome of patient 1954 (Egyptian), with 4 CHMs and 2 years of primary and secondary infertility (before the first and after the third HM), identified a variant affecting the canonical acceptor splice site of exon 27, NM_014258.4:c.2530-2A>G, in a homozygous state (Figure 2 and Supplemental Figure 5). This change was predicted to be pathogenic by the ACMG and was found in an ROH of 19.7 Mb on chromosome 20, which is in agreement with the known consanguinity between the patient’s parents (second-degree cousins). In silico analysis of the effect of this variant on *SYCP2* splicing using Human Splicing Finder (21) predicted that the c.2530-2A>G variant abolishes the splice acceptor site of exon 27 and impairs normal splicing.

In *HFM1* (helicase for meiosis 1), analysis of the exome of proband 1802 (Iranian), with a history of 2 HMs, identified a novel homozygous protein-truncating variant, NM_001017975:c.3124dupT, p.(Tyr1042Leufs*7), located in an ROH of 25 Mb on chromosome 1, which is consistent with the known consanguinity between her parents (Figure 2 and Supplemental Figure 6). This variant is predicted to be deleterious by Genomic Evolutionary Rate Profiling (GERP) (22) and CADD and pathogenic by the ACMG. The patient is from a familial case of RHM and has a sister who has had three HMs. Interestingly, the maternal aunt of the proband also experienced one HM at the age of 23, could not conceive, and then adopted a child. Analysis of additional DNA samples from this family identified the same variant in a homozygous state in the affected sister and in a heterozygous state in the 2 parents (who are third-degree cousins) and the maternal aunt, 1922. WES on blood DNA of the maternal aunt identified a second novel LP variant by the ACMG in *HFM1*, c.1159-1G>A, in a heterozygous state, that is evolutionarily highly conserved by GERP and predicted to abolish the invariant splice acceptor site of exon 8 by 2 algorithms, Splice AI (23) and Pangolin (24), with scores of 1 and 0.9 (on a scale of 0 to 1, with 1 being the most deleterious) (Figure 2 and Supplemental Figure 7). Altogether, these findings demonstrated that the maternal aunt of the proband is compound heterozygous for 2 pathogenic (P)/LP *HFM1* variants.

**Figure 3:**
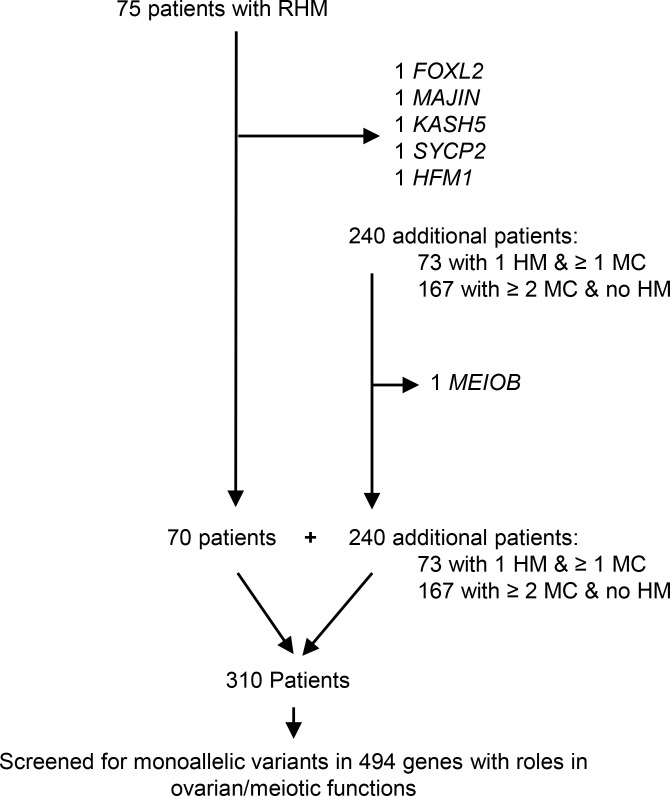
Recapitulation of the number of analyzed patients by WES from different categories. Identified genes with deleterious biallelic variants are indicated.

In an effort to identify additional patients with biallelic variants in the 5 novel genes, we analyzed the exomes of another 240 patients with related forms of reproductive loss, 73 with only 1 HM and ≥1 MC and 167 with ≥2 MCs and no HM (Figure 3). This analysis did not identify any additional patients with recessive defect in any of the 5 genes (14). However, it identified a recessive homozygous nonsense variant, NM_001163560.3:c.814C>T, p.(Arg272*), in an additional meiotic gene, *MEIOB* (meiosis specific with OB-fold), in proband 2136 (Egyptian), with a history of 6 early MCs, 3 failed intracytoplasmic sperm injection cycles, 1 HM, and low anti-Müllerian hormone (AMH) (2 times ≤0.2 ng/mL). This variant was predicted to be pathogenic by the ACMG and deleterious by SIFT and CADD and is located in an ROH of 3.5 Mb on chromosome 16, which is consistent with the known first-degree consanguinity between her parents (Figure 2 and Supplemental Figure 8) and has previously been reported in a Chinese patient with primary infertility and premature ovarian insufficiency (POI) (25).

### Patients with biallelic variants in FOXL2, MAJIN, HFM1, and SYCP2 had AnCHM.

Morphological reevaluation or DNA genotyping was possible on 10 CHMs, 5 from the patient with a *FOXL2* variant, 2 from the patient with a *MAJIN* variant, 1 from the patient with a *SYCP2* variant, 1 from the affected sister with an *HFM1* variant, and 1 from the patient with a *MEIOB* variant. Morphological analysis confirmed the diagnosis of CHM (Supplemental Figures 9–12). p57 immunohistochemistry was performed on 3 CHMs, 2 from the patient with a variant in *MAJIN* and 1 from the patient with a variant in *SYCP2*, and demonstrated a lack of p57 expression in the cytotrophoblast and stromal cells (Supplemental Figures 9 and 10). Therefore, p57 immunohistochemistry concurred with the diagnosis of CHM.

Genotype analysis using multiplex and/or simplex short tandem repeat (STR) markers was performed on 6 CHMs (*MAJIN*, *n* = 2; *HFM1*, *n* = 1; *FOXL2*, *n* = 3) and demonstrated that all of them are androgenetic monospermic (Supplemental Figures 9, 11, and 13), similar to RHMs caused by biallelic mutations in *MEI1*, *TOP6BL*, and *REC114*. Additionally, 1 molar tissue from the patient with a biallelic mutation in *MAJIN* had a maternal, non-paternal, allele at marker D13S317 from chromosome 13, indicating that this AnCHM tissue had retained 1 maternal chromosome (Supplemental Figure 9).

### A heterozygous carrier of an LP variant in MAJIN had a triploid dispermic PHM.

The mother of patient 1824, with a monoallelic P/LP variant in *MAJIN*, had experienced 1 HM at a young age (21 years). The occurrence of HM in 2 generations is extremely rare, and in most such cases, it is impossible to obtain the tissues for reevaluation and confirmation of the information provided by the probands, since many hospitals do not keep FFPE archived tissues for more than 10–15 years. We retrieved the molar tissue of the mother of patient 1824 and confirmed its diagnosis as an HM. Immunohistochemistry demonstrated positive p57 expression in the cytotrophoblast and stromal cells, which is in favor of the diagnosis of a PHM. Multiplex and simplex STR genotyping demonstrated a triploid dispermic genome (Supplemental Figure 14). Fluorescent in situ hybridization on tissue sections from this molar conception, with probes from chromosomes X, Y, and 18, confirmed its triploid genome (Rima Slim, unpublished data).

### An emerging role for deleterious biallelic variants in genes with roles in meiosis I in the formation of recurrent AnCHM.

*FOXL2* is a gene responsible for blepharophimosis, ptosis, and epicanthus inversus and premature ovarian failure 3 under the dominant and recessive modes, and some missense variants have been seen in patients with non-syndromic premature ovarian failure (without the eye abnormalities) (26, 27). *FOXL2* codes for a transcription factor and plays an important role in differentiation of granulosa cells and in downregulation of their proliferation (28).

*MAJIN* codes for a junction protein that forms a complex with TERB1 and TERB2, which together bind to telomeres and anchor them to the inner nuclear membrane components KASH5 and SUN1 (29). This attachment of chromosomes to the nuclear envelope is essential for homologous chromosome movement and synapsis. In mice, both male and female null mutants of *Kash5* (30) or *Majin* (31) are infertile. In humans, biallelic mutations in *MAJIN* have been reported in infertile males and those in *KASH5* and *TERB1* in infertile males (20, 32–34) and in females with POI (33, 35).

*SYCP2* codes for an axial/lateral element of the synaptonemal complex that is essential for meiotic homologous chromosome synapsis. Male *Sycp2*-null mice are infertile, while the females have reduced litter sizes (36). In humans, *SYCP2* P/LP variants have been reported in a heterozygous state in infertile males (37) but not in women with reproductive failure.

*HFM1* encodes a helicase for meiosis I that is essential for chromosome pairing, completion of synapsis, and recombination, and its knockout leads to male and female infertility (38). *HFM1* P/LP variants have been reported in several women with infertility and POI under both the dominant (39) and the recessive (40–43) modes, of which one had an HM (42), and in infertile men under the recessive mode (44, 45).

*MEIOB* codes for a meiosis-specific, single-stranded DNA-binding protein that is essential for repair of DSBs and homologous recombination (46, 47). Female and male *Meiob*-null mice are infertile. In humans, 9 *MEIOB* P/LP variants have been reported in men with azoospermia or oligospermia (reviewed in ref. 25) and 3 in women with POI, including a patient who had an HM, an early MC, and POI (48).

**Figure 4:**
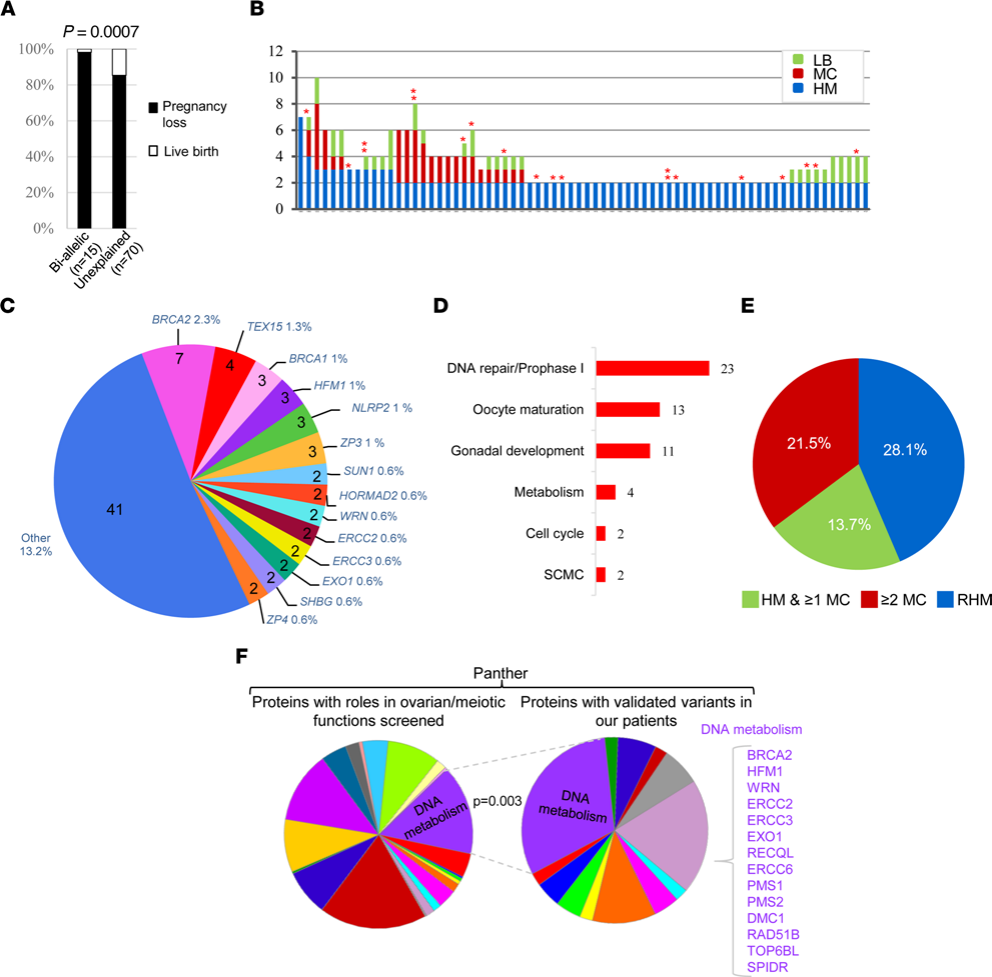
Enrichment of monoallelic P/LP variants in genes with roles in DNA metabolism. (**A**) Percentage of live births and pregnancy losses in patients negative for causative biallelic variants. (**B**) Detailed reproductive history of the 70 patients with RHM shown in **A**. Each patient is represented by a vertical bar on the *x* axis, and the number of each type of her pregnancy outcomes on the *y* axis. Asterisks denote the number of identified P/LP variants in the patient. (**C**) Proportional contribution of genes to the genetic susceptibility of the 310 analyzed patients. Numbers inside the pie indicate the number of patients with P/LP variants in each gene, and percentages outside the pie indicate the proportion of patients with P/LP variants in a given gene among the 310 patients. (**D**) Roles and functions of genes with P/LP variants based on GeneCards and PubMed. The numbers of variants in genes with the indicated functions are shown on the right of the bars. (**E**) The frequencies of P/LP variants in the 3 categories of patients. (**F**) Panther analysis showing a significant enrichment of monoallelic P/LP variants in the category of DNA metabolism in our patients while proteins from this category accounted for a smaller percentage in our input list. Statistical analysis was performed using 2-tailed Fisher’s exact test in **A** and χ^2^ test in **F**. *P* < 0.05 was considered significant.

We previously identified biallelic deleterious variants in 3 other meiosis I genes, *MEI1*, *TOP6BL*, and *REC114*, in patients with AnCHM (6). *MEI1*, *TOP6BL*, and *REC114* play roles in DSB formation, which facilitates synapsis. Taken together, the 3 previously identified genes and the 5 meiosis I genes identified in this study (*MAJIN*, *KASH5*, *SYCP2*, *HFM1*, and *MEIOB*) suggested a major role of defects in meiotic synapsis and recombination in the formation of AnCHM. Comparing the reproductive outcomes of all our patients with androgenetic RHM and recessive P/LP variants in the 9 genes (*n* = 15 patients) with those of patients who had remained negative (*n* = 70) for biallelic P/LP variants revealed that the former had a significantly more severe phenotype with more reproductive losses and fewer live births (Figure 4A). The 15 patients with biallelic P/LP variants had only 1 live birth (in the patient with a missense variant in *FOXL2*) and a total of 62 pregnancy losses (HM and MC), while the 70 patients with unexplained genetic etiology had a total of 39 live births and 198 pregnancy losses (2-tailed Fisher’s exact *P* value = 0.0007) (Figure 4A). These data demonstrated that the 70 patients with genetically unexplained etiology had, globally, milder phenotypes than those with biallelic mutations, suggesting milder genetic defects and possibly different modes of transmission of their defects. Moreover, these 70 negative patients did not have a large number of pregnancy losses (Figure 4B) as do patients with biallelic *NLRP7* or *KHDC3L* mutations, which has been a consistent observation in our laboratory for the past decade. Indeed, the 70 negative patients had a total of 237 conceptions (HM, MC, and live birth), an average of 3.38 conceptions per patient, while our 130 patients with biallelic *NLRP7* or *KHDC3L* mutations had a total of 649 conceptions (Rima Slim, unpublished data), an average of 4.99 conceptions per patient (χ^2^ test *P* value = 0.019). This difference suggested that perhaps some of the 70 negative patients may have an undiagnosed subclinical secondary infertility or difficulties conceiving, which has been previously reported in patients with common sporadic HM (49). Third, the mother of one patient, who is of European origin and a heterozygous carrier of 1 LP variant in *MAJIN*, had 1 PHM at the age of 21. At this age, the frequency of sporadic PHM among Europeans is estimated at 1 in 1,488 pregnancies (1), which makes it unlikely that her PHM was only by chance. Altogether, these data suggested that carriers of monoallelic P/LP variants in *MAJIN*, and perhaps also in other meiotic genes, may underlie the milder phenotype of the remaining 70 negative patients.

### Enrichment of monoallelic variants in meiosis I genes in an additional 240 patients with milder phenotypes.

To investigate whether monoallelic variants in genes with roles in meiosis and ovarian functions underlie the genetic susceptibility to RHM in patients with unexplained etiology, we searched PubMed and the Panther (50) database for such genes and established a list of 494 genes (Supplemental Table 1) (51, 52) that we screened for the presence of monoallelic deleterious variants in the exomes of the 70 patients with RHM. None of these patients were from a familial case of RHM or had any HM with a diploid biparental genome, but several of them did not have any HM tissues available for genotyping or morphological reevaluation. We then extended this analysis to the exomes of 240 additional patients, 73 with 1 HM and ≥1 MC and 167 with ≥2 MCs and no HM, all of whom did not have plausible candidate variants under the recessive mode. To enrich for variants with clear severe functional impacts, we only looked for loss-of-function (LoF) variants that lead to stop gain or to frameshift due to small deletions or insertions, or affect the invariant splice sites, and were rare in our in-house controls and in the Genome Aggregation Database (gnomAD). Selected variants were evaluated according to the ACMG guidelines using VarSome, and only those that were predicted to be pathogenic, LP, or VUS with pathogenic or LP (variants originally scored as VUS but that have been revised recently by VarSome to VUS pathogenic or VUS LP) were validated by Sanger sequencing and segregated in available family members.

This analysis led to the identification of 77 different P/LP variants in 55 genes in 68 patients (21.8%) (Supplemental Table 2). The highest load of P/LP variants in our 310 patients was in *BRCA2*, with 5 different P/LP variants, 2 frameshifts, 2 stop codons, and 1 invariant splice that leads to the skipping of exon 2 containing the translation start site, in 5 patients of different ethnic origins (European, African, Indian, and Latino) (Figure 4C, Supplemental Table 2, and Supplemental Figures 15 and 16). An additional 2 patients, 1985 and 1624, had an identical extended splice site variant, c.9501+3A>T, that impairs the splicing of *BRCA2* in a lymphoblastoid cell line from the patient and leads to the skipping of exon 25 in a fraction of transcripts (Supplemental Table 2 and Supplemental Figure 16) and is in agreement with a previous report (53). These 2 patients are of European and Latino origins, in which the minor allele frequency of this variant is 0.0002 and 0.000174, respectively (gnomAD) (54). The other most mutated genes were *TEX15* followed by *BRCA1*, *HFM1*, *NLRP2*, and *ZP3* (Figure 4C, Supplemental Figures 17–19, and Supplemental Table 2). Seven other genes, *SUN1*, *WRN*, *ERCC2*, *ERCC3*, *EXO1*, *SHBG*, and *HORMAD2*, each had 2 different variants, and *ZP4* had 1 variant in 2 patients from different ethnic origins. Forty-one other genes each had 1 variant in only 1 patient (Figure 4C and Supplemental Table 2). Two patients had variants in 3 genes, while 9 patients had variants in 2.

Analysis of the functions of genes with these variants using GeneCards and PubMed searches showed that the highest number of variants were in genes with roles in DNA repair and prophase I, followed by genes with roles in oocyte maturation, gonadal development, metabolism, cell cycle, and the SCMC (Figure 4D). The highest frequencies of patients with at least one P/LP variant were among patients with RHM (28.1%) followed by patients with ≥2 MCs and no HM (21.5%) and then patients with 1 HM and ≥1 MC (13.7%) (Figure 4E). It is notable that among the patients with ≥2 MCs and no HM and P/LP variants, 11 (7%) had primary or secondary infertility and sought the help of assisted reproductive technology (ART) services (Supplemental Table 2). For a few patients, in addition to the recurrent pregnancy loss, the referral included suspected poor ovarian functions or oocyte quality following in vitro fertilization.

### Comparison between our patients and the general population in gnomAD.

Comparing the minor allele frequencies of P/LP LoF variants in our patients and in the general population of gnomAD (807,162 unrelated subjects, 730,947 exomes, and 76,215 genomes from diverse ancestries, v4.1.0) revealed that P/LP LoF variants in *BRCA2* (excluding the 2 patients with the extended splice site variant) and *ZP3* were significantly more frequent in our patients (2-tailed Fisher’s exact *P* value = 0.0010 and 0.0009, respectively; Supplemental Table 3). Of these 2 genes, the most intolerant for LoF variants is *ZP3*, with a probability of being loss of function intolerant (pLI) of 0.95. There are no publicly available exomes of patients with RHM. One study analyzed the exome of 51 patients with sporadic HM and matching controls (55). Screening its public data for genes with a single deleterious variant in our 494 ovarian and meiosis genes identified P/LP variants according to the ACMG in 7 genes, *NOBOX*, *TEX15*, *HFM1*, *SOX8*, *REC8*, *FANCL*, and *ERCC6*, only in the patient group.

### Enrichment of variants in DNA metabolism proteins.

We next investigated whether our patients have an enrichment of P/LP monoallelic variants in genes coding for a specific class of proteins in Panther (50). Of the 494 genes we screened in our patients, 482 were classified by Panther. The analysis of our 482 genes and those with validated P/LP variants in our patients (Supplemental Table 2) revealed that genes coding for the protein class of DNA metabolism (PC00009) are significantly enriched in our patients (Figure 4F and Supplemental Figures 20–22). Genes coding for this protein class accounted for 11% in our input list (53 of 482) and for 25% of genes with validated variants in our patients (14 of 57) (*P* = 0.003). Members of this enriched protein class in our patients are *BRCA2*, *HFM1*, *WRN*, *ERCC2*, *ERCC3*, *EXO1*, *RECQL*, *ERCC6*, *PMS1*, *PMS2*, *DMC1*, *TOP6BL*, and *SPIDR*, all of which have roles in changing DNA conformation or in DNA excision and repair, which are essential steps for meiosis I progression (Figure 4F). Altogether, these data indicated that monoallelic P/LP variants in genes with roles in early stages of meiosis I are enriched in patients who have milder phenotypes and most likely confer on these patients a genetic susceptibility for RHM, recurrent MCs, and infertility.

### Hfm1-null female mice display meiotic synapsis failure in their oocytes.

**Figure 5:**
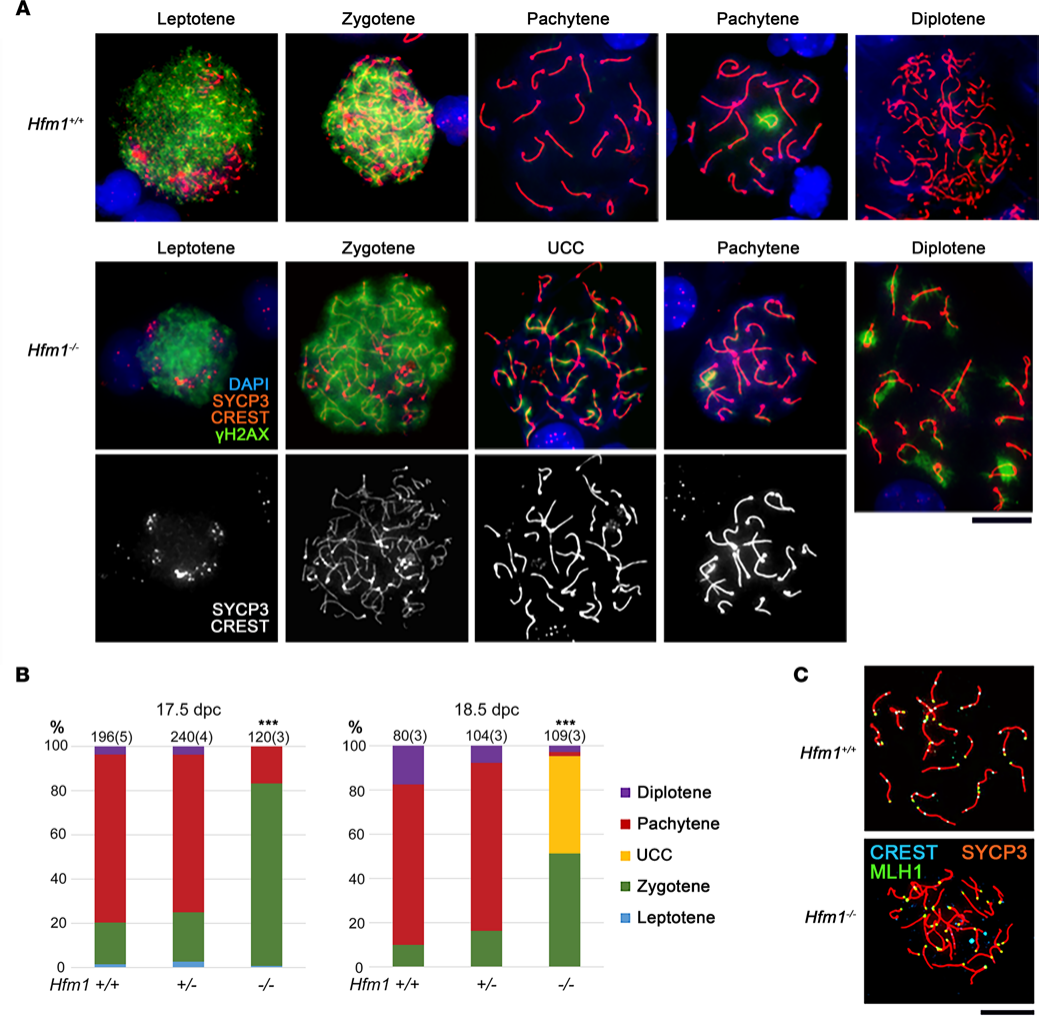
Meiotic prophase I progression in *Hfm1^+/+^*, *Hfm1^+/–^*, and *Hfm1^–/–^* females. (**A**) Meiotic prophase I (MPI) substages identified in the microspread ovarian cells stained with immunofluorescence for a component of the synaptonemal complex (SYCP3), centromere (CREST), DSB (γH2AX), and DNA (DAPI). UCC, unsynapsed condensed chromosomes. (**B**) Percentages of MPI substages at 17.5 and 18.5 dpc. The total number of oocytes examined is given on the top of each column along with the number of females (in parentheses). ***Significant difference between *Hfm1^–/–^* and either *Hfm1^+/+^* or *Hfm1^+/–^* females at *P* < 0.001 by χ^2^ test. (**C**) MLH1 foci at the late pachytene stage indicating the crossover sites. Scale bar: 20 μm.

Biallelic P/LP variants in *MAJIN* and *HFM1* were the first to be identified in our patients, and *HFM1* variants were observed in a familial case consisting of 3 affected members. We therefore focused on *Hfm1*-null mice (38) to model the mechanism of AnCHM formation. *Hfm1^–/–^* males are reported to be healthy but sterile as a result of incomplete meiotic crossover and elimination of spermatocytes (38). The authors of this study also showed that *Hfm1^–/–^* females are sterile with very few follicles in their ovaries at 45 days postpartum (dpp). However, they did not examine the defects in the oocytes or determine the meiotic stage at which the oocytes are lost. We therefore examined the meiotic synapsis in microspread oocytes from *Hfm1^+/+^*, *Hfm1^+/–^*, and *Hfm1^–/–^* fetal ovaries at 17.5 and 18.5 dpc. In the wild-type ovary, meiotic prophase I is divided into 4 substages: leptotene, zygotene, pachytene, and diplotene (Figure 5A). At the leptotene stage, a synaptonemal complex component, SYCP3, accumulates along the chromosome cores while numerous DSBs form along the DNA, visualized by γH2AX. At the zygotene stage, homologous chromosomes begin to synapse between the SYCP3 cores. At the pachytene stage, chromosome synapsis is completed with repair of DSBs and removal of γH2AX. When a pair of chromosomes fails to synapse, the unsynapsed chromosomes are locally covered with γH2AX cloud(s). At the diplotene stage, homologous chromosomes begin to separate except at the crossover sites.

In both *Hfm1^+/+^* and *Hfm1^+/–^* ovaries, 70%–80% of oocytes reached the pachytene stage at 17.5 dpc, and 10%–20 % advanced to the diplotene stage at 18.5 dpc (Figures 5, A and B). By contrast, in *Hfm1^–/–^* ovaries at 17.5 dpc, only 18% of oocytes reached the pachytene stage and all the others were still at the zygotene stage (*P* < 0.001 by χ^2^ test). Very few of the pachytene oocytes showed complete synapsis, and most oocytes retained some γH2AX clouds to varying extents (Figure 5A). At 18.5 dpc, approximately 40% of oocytes showed unsynapsed condensed chromosomes that were often univalents. Where chromosomes were synapsed, MLH1 foci, which mark crossover sites, were absent in *Hfm1^–/–^* oocytes (Figure 5C), in agreement with the findings in *Hfm1^–/–^* spermatocytes (38). Thus, *Hfm1^–/–^* oocytes lack the crossovers between homologous chromosomes, which are essential for proper chromosome segregation at the first meiotic division.

### Majin^–/–^ and Hfm1^–/–^ mice display oocyte loss and severe ovarian dysgenesis before puberty.

**Figure 6:**
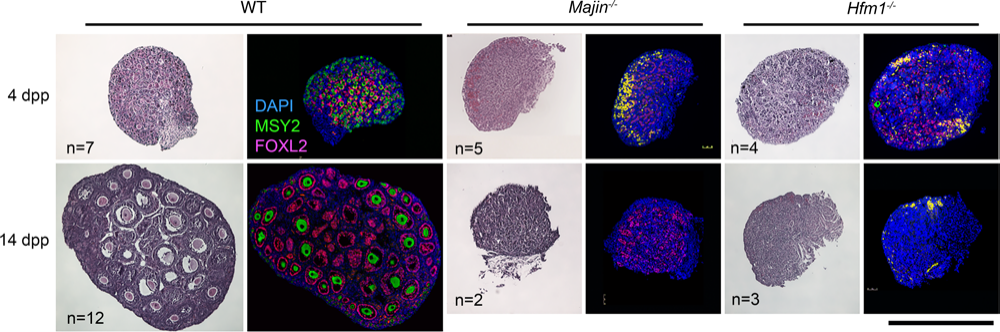
Histological sections of postnatal ovaries from wild-type, *Majin^–/–^*, and *Hfm1^–/–^* females. H&E (left) or immunofluorescence staining for MSY2 and FOXL2 with DAPI (right). MSY2 is a marker for oocytes at or beyond the diplotene stage. FOXL2 is a marker for granulosa cells. Blood cells are seen in yellow due to autofluorescence. Scale bar: 500 μm.

In histological sections of wild-type ovaries at 4 dpp, follicles were formed by surrounding individual oocytes with granulosa cells, visualized by immunofluorescence staining of MSY2 and FOXL2, respectively (Figure 6). By contrast, in *Majin^–/–^* and *Hfm1^–/–^* ovaries, very few oocytes remained, while FOXL2-positive granulosa cells were scattered. Blood cells often occupied the areas where the oocytes had been depleted in both null ovaries. It is well known that oocytes with failure in chromosome synapsis or DSB repair are eliminated by a checkpoint during the neonatal period (56, 57). Therefore, the loss of oocytes in *Hfm1*-null ovaries appears to be comparable to deficiencies in other components essential for meiotic synapsis (58–60). At 14 dpp, follicles at various stages were formed in wild-type ovaries, whereas follicles were rarely seen in either *Majin^–/–^* or *Hfm1^–/–^* ovaries, although FOXL2-positive cells were still present. At 21–25 dpp, despite the major oocyte loss, follicles were occasionally found in *Hfm1^–/–^* ovaries, which were significantly smaller than wild-type ovaries (Supplemental Figure 23). *Majin^–/–^* ovaries were even smaller and were not easily identifiable (Supplemental Figure 23), preventing us from pursuing further studies on their oocytes.

### Oocytes from Hfm1^–/–^ females show delayed meiotic progression and difficulty in separating chromosomes.

**Figure 7:**
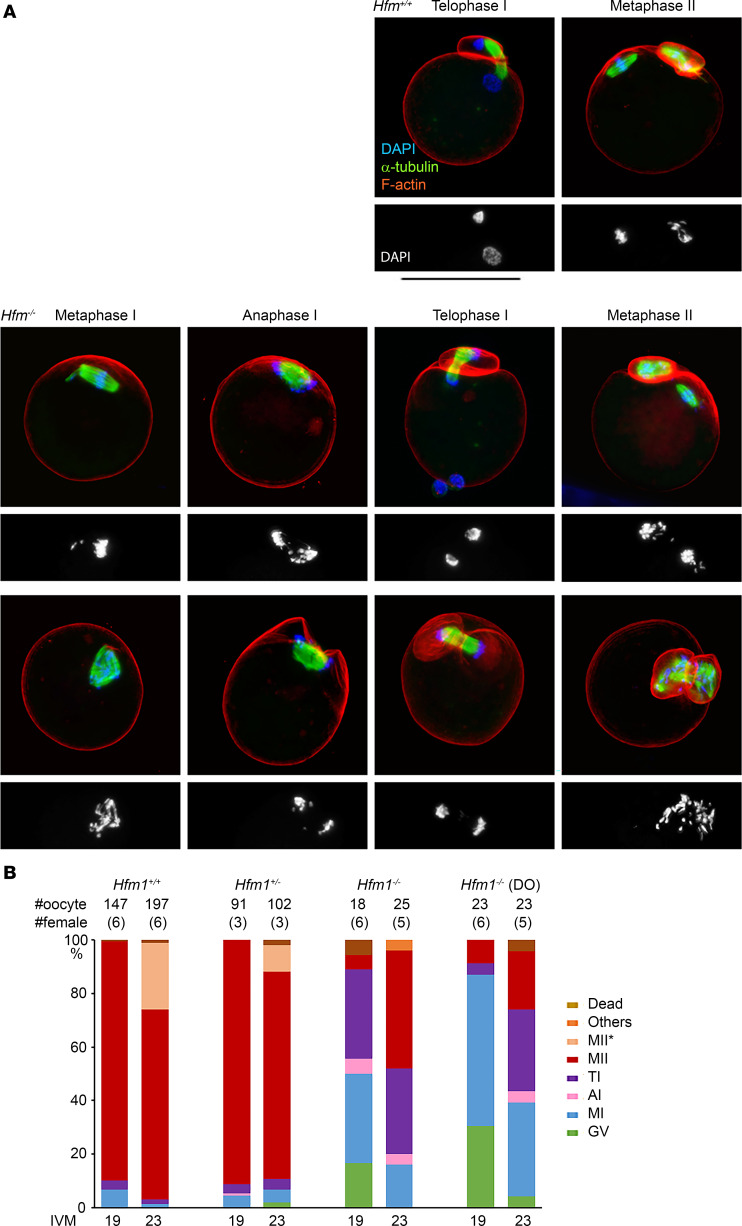
In vitro meiotic progression of oocytes from *Hfm1^+/+^* and *Hfm1^–/–^* females. (**A**) Confocal microscopy images after culture for 19–23 hours. DAPI staining alone is shown beneath each merged image. *Hfm1^+/+^*, representative images of the majority of oocytes; *Hfm1^–/–^* top, images observed that are closest to those from *Hfm1^+/+^* females; *Hfm1^–/–^* bottom, examples of anomalies that were rarely seen in the oocytes from *Hfm1^+/+^* females. Scale bar: 100 μm. (**B**) Percentage of oocytes in each meiotic stage. DO, spontaneously denuded oocytes before culture. MII* indicates that the spindle resembles MII but no polar body or chromosomes are seen outside the oocyte.

**Figure 8:**
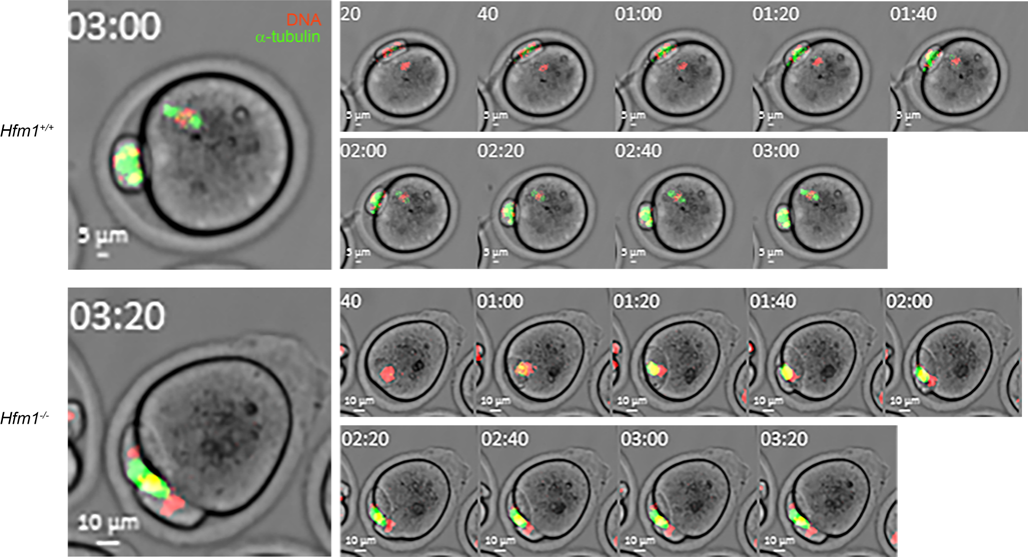
Live imaging of meiotic progression in the oocytes from *Hfm1^+/+^* and *Hfm1^–/–^* females. Staining of DNA (red) and α-tubulin (green) with phase contrast. Left: The oocytes at the end of imaging at a higher magnification. Time-lapse imaging started after 19 hours of IVM. (See also Supplemental Videos 1 and 2.)

After females at 22–25 dpp were injected with gonadotropins, a much smaller number of germinal vesicle–stage oocytes surrounded by cumulus cells (cumulus-oocyte complexes [COCs]) were recovered from *Hfm1^–/–^* females (on average 3.9 ± 0.6 COCs per female from 18 mice) compared with *Hfm1^+/+^* females (on average 36.3 ± 2.6 COCs per female from 15 mice). When oocytes in COCs were subjected to in vitro maturation (IVM) for 19 hours, 89% of *Hfm1^+/+^* oocytes reached the second metaphase (MII) stage, while only 1 of 18 *Hfm1^–/–^* oocytes did so (Figure 7, A and B). Thirty-three percent of *Hfm1^–/–^* oocytes reached the telophase I (TI), but 17% and 33% of oocytes remained at the germinal vesicle and metaphase I (MI) stages, respectively. When IVM was extended for 23 hours, 44% of *Hfm1^–/–^* oocytes reached the MII stage, while 32% remained at the TI stage (*n* = 25). Since the number of COCs recovered from *Hfm1^–/–^* females was limited, spontaneously denuded oocytes were also subjected to IVM. Such oocytes are known to have a lower competence for meiotic progression (61–63). In agreement, a larger percentage (35%) of denuded oocytes from *Hfm1^–/–^* females remained at the MI stage up to 23 hours of IVM (Figure 7B). Nonetheless, other oocytes followed similar meiotic progression to those in COCs by reaching the TI and MII stages (Figure 7B). Representative images of the oocytes during meiotic progression are shown in Figure 7A. In both types of oocytes (with or without COCs) from *Hfm1^–/–^* females, single spindles were formed at the MI stage and segregated their chromosomes into 2 poles at the anaphase I (AI) and TI stages; however, chromosomes were often seen scattered over the spindles, which were positioned parallel to the ooplasmic membranes. In some of the oocytes that reached the MII stage, all the chromosomes were extruded into the first polar body(ies) (formation of multiple polar bodies was common in *Hfm1^–/–^* MII oocytes). An example of an oocyte that extruded all its chromosomes into the first polar body is shown in Figure 7A, and another is shown with time-lapse imaging in Figure 8 and Supplemental Video 1. A total of 4 out of 29 (14%) MII oocytes from *Hfm1^–/–^* females extruded all their chromosomes into polar bodies. Note that most oocytes from *Hfm1^+/+^* females were already at the MII stage when live imaging was started and formed MII spindles before or during the imaging period (Figure 8 and Supplemental Video 2).

## Discussion

In this study, we identified 8 deleterious biallelic variants in 6 novel genes, *FOXL2*, *MAJIN*, *KASH5*, *SYCP2*, *MEIOB*, and *HFM1*, in 6 unrelated patients with recurrent HMs and MCs, including one from a familial case of 3 affected members. *FOXL2* codes for a transcription factor that regulates granulosa cell differentiation, while the remaining 5 genes play roles in early stages of meiosis I and all the 6 genes have established roles in POI. Ten molar tissues from the patients with P/LP biallelic variants in *FOXL2* (*n* = 4), *MAJIN* (*n* = 2), *SYCP2* (*n* = 1), *HFM1* (*n* = 1), and *MEIOB* (*n* = 1) were available for morphological reevaluation and four for immunohistochemistry with p57, and their diagnosis was confirmed as CHM. Of the 10 CHMs, 6 were genotyped and found to be androgenetic monospermic. These data, taken together with the roles we previously demonstrated of 3 other meiosis I genes, *MEI1*, *TOP6BL*, and *REC114*, in the causation of recurrent AnCHM (6), revealed an emerging major role of meiosis I defects in the genesis of AnCHM.

Among patients with RHM, those with biallelic variants (*n* = 15) had significantly more severe reproductive outcomes than the 70 patients who have remained negative for biallelic variants, suggesting that the latter patients, or at least some of them, have milder genetic defects. We hypothesized that monoallelic P/LP variants in ovarian and meiotic genes could underlie their genetic susceptibility to RHM and tested this hypothesis by screening their exomes for monoallelic variants in a total of 494 genes with known roles in ovarian and meiotic functions. Because our cohort of patients with RHM is relatively small owing to the scarcity of this condition, we extended our analysis to patients with other forms of reproductive loss, which included 73 patients with 1 HM and ≥1 MC and 167 patients with ≥2 MCs and no HM. To enrich for variants with deleterious functional impact on the proteins, we looked only at variants that lead to protein truncation and are predicted to be P/LP by the ACMG that we segregated in the patient family members. The most mutated genes were *BRCA2*, *TEX15*, *HFM1*, *NLRP2*, and *ZP3*, and 2 of them, *BRCA2* and *ZP3*, reached statistical significance when the frequencies of their P/LP variants were compared with those of all protein-truncating variants in these genes in the general population analyzed by gnomAD (v4.1.0) (2-tailed Fisher’s exact *P* = 0.001 for both genes). Carriers of validated monoallelic P/LP variants in *BRCA2* and *ZP3* were 6.7 and 16 times more frequent, respectively, in our patients than in carriers of all protein-truncating variants in these 2 genes in gnomAD. We believe that these relative frequencies are underestimated, for the following reasons. First, all our variants were validated by Sanger sequencing, while those listed in gnomAD were not. Second, our analysis included only protein-truncating variants predicted to be P/LP, while not all protein-truncating variants in gnomAD have been classified. Third, the subjects analyzed in gnomAD may include women with POI,since these women are healthy and only some of them may manifest POI if they attempt to reproduce at an advanced age. Our data on *BRCA2* have been obtained on patients from various ethnic groups referred to our laboratory from different collaborators and countries and consequently are not subject to population stratification but rather reflect an enrichment of such *BRCA2* variants in patients with reproductive failure. These data are in line with those from 2 recent studies documenting higher frequencies of P/LP variants in *BRCA2* in infertile women from the UK and China than in the general populations (64, 65). *BRCA2* P/LP variants have been shown to impair the cellular response to DNA damage owing to failure of the recruitment of RAD51, a key protein for DNA repair, to DSBs (66, 67). Since DSBs are harmful for the cells, this will activate apoptotic pathways and lead to the death of the oocytes and consequently diminished ovarian reserve. In addition, patients with hereditary breast and ovarian cancers and P/LP variants in *BRCA2* have been shown to have significantly lower AMH levels than controls (68), and 2 of our patients with P/LP variants in *BRCA2* had low AMH (patients 1093 and 1985; Supplemental Table 2). Monoallelic pathogenic variants in *ZP3* have been documented to cause female infertility due to oocyte maturation arrest that may also manifest as empty follicle syndrome, zona-free oocytes, or zona-thin oocytes in patients attempting ART (reviewed in ref. 69).

Comparing the protein classes of the screened genes and those that had validated monoallelic P/LP variants in our patients using Panther revealed significant enrichment for variants in genes coding for the protein class of DNA metabolism (*P* = 0.003), which includes proteins involved in modifying the structure of DNA such as helicases and topoisomerases (HFM1, WRN, and RECQL) and proteins involved in DNA repair (BRCA2, PMS2, PMS1, SPIDR, and EXO1), all of which have established roles in POI. For patient counseling, it is important to keep in mind that patients with monoallelic variants in these genes can conceive and have healthy children; however, they are at higher risk for infertility, POI, and reproductive loss than women from the general population as shown in this study and others (64, 65). With advanced maternal age, many of these patients ultimately turn to medically assisted reproductive technologies, from which some of the patients analyzed in this study and others were recruited (64, 65).

**Figure 9:**
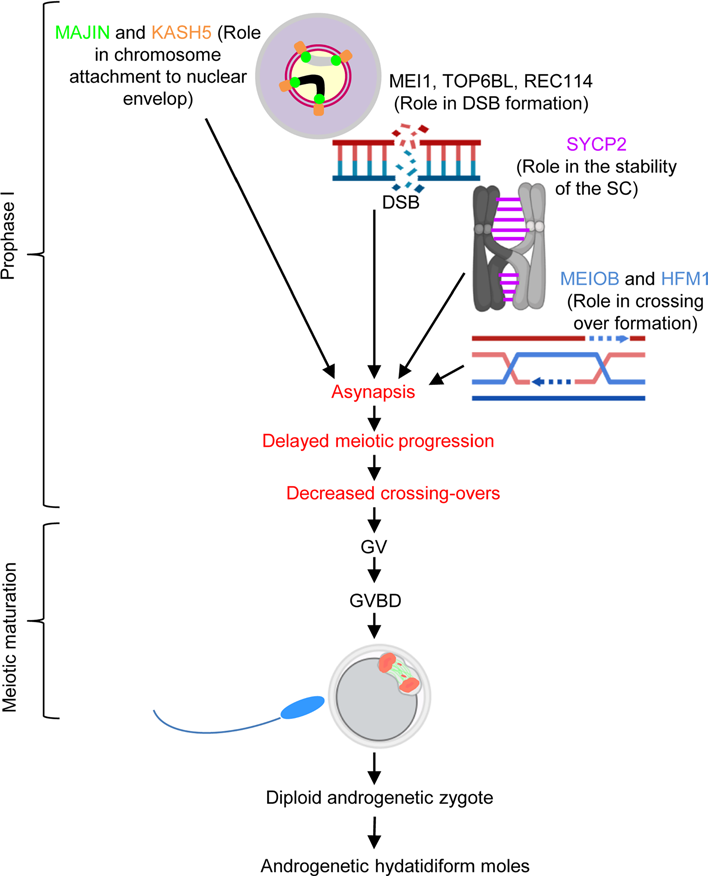
Schematic representation of the roles of the 5 meiotic prophase I genes and our hypothesis on the formation of AnCHM. GV, germinal vesicle; GVBD, germinal vesicle breakdown.

Mice carrying null mutations of the 6 genes, *Foxl2*, *Majin*, *Kash5*, *Sycp2*, *Hfm1*, and *Meiob*, have been previously examined. FOXL2 is essential for granulosa cell differentiation and proliferation, as well as ovarian maintenance and function (70). Therefore, its impairment may affect indirectly the meiotic maturation of oocytes, and may consequently lead to molar pregnancies. Since the identified missense is classified as VUS, this single variant does not allow conclusions regarding the causal role of *FOXL2* in RHM, which remains to be validated in future studies and patients. The proteins coded by the other 5 genes, despite their involvement in distinct mechanisms, all play essential roles in homologous chromosome synapsis and crossing over in the oocytes during fetal development (Figure 9). MAJIN and KASH5 mediate the attachment of telomeres to the nuclear envelope, which is critical for homologous chromosome search and synapsis (71–73). SYCP2 is a component of the synaptonemal complex that supports chromosome synapsis (36, 74). HFM1 and MEIOB play roles in the completion of synapsis and meiotic recombination in spermatocytes (38, 46). Our current study demonstrates a key role of HFM1 in chromosome synapsis in oocytes. The observation of entirely unsynapsed chromosomes in *Hfm1-*null oocytes is reminiscent of *Majin*- and *Sun1*-null oocytes and in agreement with a previous report that HFM1 is required for chromosome movement during homolog search (75). It is known in mouse oocytes that the presence of unsynapsed chromosomes and unrepaired DSBs beyond the pachytene stage provokes the CHEK2-dependent checkpoint to eliminate these cells, resulting in a small ovarian reserve (56, 76). However, to our knowledge, it is unknown whether a similar checkpoint mechanism operates in human oocytes. The association of their homologs with POI in both species is in favor of a common mechanism for oocyte elimination. In the *Hfm1-*null mice, the surviving oocytes formed follicles and resumed the meiotic progression in culture. We anticipated that the oocytes would be arrested at the MI stage because the absence of crossovers between the homologous chromosomes would provoke the spindle assembly checkpoint to arrest meiotic progression (77–79). To our surprise, however, most oocytes passed the MI stage and reached the AI and TI stages. Furthermore, while meiotic spindles were positioned perpendicular until a half set of chromosomes was extruded into the polar bodies in wild-type oocytes, the spindles were more often positioned parallel to the ooplasmic membrane in the *Hfm1-*null oocytes, and F-actin caps formed and engulfed both sets of chromosomes at the spindle poles (Figure 7A, bottom). Subsequently, formation of 2 or more polar bodies containing all chromosomes was frequently seen in the MII oocytes from *Hfm1^–/–^* females. Thus, a failure in chromosome alignment on the MI spindle rather caused a delay or arrest at TI, and of the oocytes that managed to reach MII, 14% extruded all the chromosomes into the first polar bodies. It is remarkable that the endpoint of *Hfm1*-null oocytes was similar to that of *Mei1*-null oocytes as we previously reported (6) even though they do not follow exactly the same course of meiotic progression.

POI is defined by the cessation of menses for at least 6 months and high serum levels of follicle-stimulating hormone (FSH) as well as low antral follicle counts and low serum levels of AMH. Among the 3 categories of reproductive loss we analyzed, the highest frequency of validated monoallelic P/LP variants in ovarian and meiotic genes was observed in patients with RHM (28.1%), yet none of the patients had been diagnosed with POI at the time of referral and only 2 had sought ART services (patients 1118 and 1601). Advanced maternal age is the strongest risk factor for HM, as has been replicated in all studies and populations (1, 80). This risk increases sharply after the age of 40 and is approximately 100 times higher at the age of 50 than at the age of 25 (1). In fact, of all forms of reproductive loss, the pathology of HM, and mainly AnCHM, is the most sensitive to maternal age and displays the highest increase in risk with advanced maternal age. Our data link POI to the pathology of RHM and provide an explanation for the increased incidence of common AnCHM with advanced maternal age. In addition, all HMs, sporadic or recurrent, are caused by oocyte defects, while MC is a more heterogeneous entity and may result from various defects, such as uterine and endometrial defects, reproductive tract infections, and undiagnosed defects in the male gametes. Therefore, the RHM phenotype has reduced the genetic heterogeneity of the category of patients with RHM and enriched for those with oocyte defects who are more likely to have a genetic susceptibility in ovarian and meiosis I genes. The data from our study suggest that recurrent AnCHM may be a sign of accelerated ovarian aging or a milder form of POI before its manifestation by clinical and laboratory tests. Our study calls for the necessity of evaluating the ovarian functions and reserve of such patients.

## Methods

### Sex as a biological variable.

Females were recruited or studied, because the study addressed pregnancy failure in females. Males were studied only if they were the partners, siblings, or parents of affected females. Some of the data are relevant to both sexes.

### Patient samples.

Written informed consent was obtained from all participants who provided blood, saliva, or products of conception. The patients analyzed in this study included 75 unrelated patients with RHM, 73 patients with 1 HM and ≥1 non-molar miscarriage (1 HM and ≥1 MC), and 167 patients with recurrent miscarriage (≥2 MCs) and no HM (Figure 3). Patients with RHM were recruited from various international collaborators, while patients with 1 HM and ≥1 MC and patients with ≥2 MCs and no HM were recruited mostly from 2 clinics in Montreal, the Repeated Pregnancy Loss Clinic at the McGill University Health Centre and Le Réseau des Maladies Trophoblastiques du Québec (81). FFPE archived products of conception were retrieved for analyses from various national and international pathology laboratories.

### Mutation analysis.

Patients with RHM were screened by Sanger sequencing for mutations in *NLRP7* (14) and *KHDC3L* as previously described (82), and those who were found to be negative for biallelic mutations in the 2 genes were analyzed by WES.

### DNA extraction and genotyping.

Genomic DNA was isolated from whole blood cells using the Flexigene DNA Kit (QIAGEN), from saliva using the PrepIT.L2P kit (DNA Genotek), or from FFPE tissues using the QIAamp DNA FFPE Tissue Kit (QIAGEN) according to the manufacturers’ instructions. Multiplex and simplex short tandem repeat genotyping was performed as previously described (2).

### Histopathology and genotype analyses of products of conception.

Sectioning, H&E staining, and p57 immunohistochemistry were performed according to standard methods as previously described (2, 14).

### Library preparation and WES analyses.

Five hundred nanograms peripheral blood leukocyte DNA from patients was captured with either Roche Nimbelgen SeqCap EZ Human Exomes or MedExomes capture kits and then sequenced with paired-end 100 bp reads on Illumina HiSeq 6000. Sequence reads were mapped to the human reference genome (hg19) with Burrows-Wheeler Aligner (5) (v0.7.17), processed, and analyzed as previously described (6).

For the search for enrichment of P/LP monoallelic protein-truncating variants, we established a list of 494 genes with known roles in ovarian and meiotic functions based on several review papers (Supplemental Table 1) (52, 83–93). We combined all variants identified in our patients into a single Excel sheet that we filtered for protein-truncating variants that had a minor allele frequency less than 0.005 in gnomAD, were rare in our in-house exomes, and were predicted to be P, LP, VUS with P (VUS P) or with LP (VUS LP) according to the ACMG guidelines using VarSome (16). We then downloaded all protein-truncating variants in the general population of gnomAD (v4.1.0), filtered out variants that were benign, likely benign, or VUS variants, and kept all unclassified protein-truncating variants in gnomAD. The frequency of protein-truncating variants for each gene was calculated by counting the number of all protein-truncating alleles and dividing it by the average number of all alleles. These frequencies were compared with the number of protein-truncating alleles divided by the total number of alleles in our patients.

### Mice.

Mice carrying heterozygous null mutation of *Majin* (048518-UCD) and *Hfm1* (OST347241) were purchased from MMRRC (Mutant Mouse Resource & Research Centers, Davis, California, USA) and Texas A&M Institute for Genomic Medicine (TIGM; College Station, Texas, USA), respectively. Both strains were maintained on the C57BL/6J background (The Jackson Laboratory) and crossed to produce homozygous null mice. *Majin* and *Hfm1* genotyping were carried out according to the MMRRC and TIGM protocols. Mice were kept in a temperature- and light-controlled room with food ad libitum.

### Immunofluorescence staining of microspread ovarian cells.

Mouse ovaries were isolated at 17.5 and 18.5 dpc and individually processed for microspread cell preparations as previously described (94). The cells on histology slides were immunofluorescence-stained (IF-stained) to identify germ cells (GCNA1), an axial element of the synaptonemal complex (SYCP3), centromeres (CREST), and either DNA DSBs (γH2AX) or crossovers (MLH1). The primary and secondary antibodies used are listed in Supplemental Table 4. After IF staining, slides were washed and mounted in Prolong Antifade mounting medium containing 4′,6-diamidino-2-phenylindole dihydrochloride (DAPI) (Molecular Probes). Fluorescent signals were captured and analyzed under an epifluorescence microscope (Leica Microscope System DM6000B).

### H&E and immunofluorescence staining of ovarian sections.

Mouse ovaries at 4 and 14 dpp were fixed in 2% formaldehyde in microtubule stabilizing buffer (95), embedded in paraffin, and sectioned at 5 μm. A slide containing 4–6 serial sections from each ovary was stained with H&E according to standard methods. Another slide from each ovary was deparaffinized and subjected to antigen retrieval as previously described (96), followed by IF staining to identify oocytes (MSY2) and granulosa cells (FOXL2). The primary and secondary antibodies are listed in Supplemental Table 4. After IF staining, the slides were washed, mounted as described above, and examined under light and epifluorescence microscopy.

### IVM of oocytes.

Female mice at 23–25 dpp were intraperitoneally injected with 10 IU equine chorionic gonadotropin (eCG; MilliporeSigma), and 46 hours later, germinal vesicle–stage oocytes surrounded with cumulus cells (COCs) were collected and cultured for up to 23 hours as previously reported (97). The stage of oocytes was examined after cumulus cells were stripped off. Some oocytes were fixed in 2% paraformaldehyde and IF-stained with anti–α-tubulin antibody conjugated with Alexa Fluor 488 (Supplemental Table 2) and 5 IU/mL Rhodamine-Phalloidin-TRITC (MilliporeSigma) for 1 hour at room temperature. After washing, the oocytes were mounted in Vectashield containing DAPI (Vector Laboratories) on histology slides. Fluorescence was visualized with a Zeiss LSM780-NLO laser scanning confocal microscope with IR-OPO lasers using a ×20 objective (Plan-Apochromat ×20/0.8 WD = 0.55) at the Molecular Imaging Facility of the Research Institute of the McGill University Health Centre (RI-MUHC).

### Live imaging of oocytes.

Some oocytes in COCs were subjected to IVM for 18.5 hours as above, stripped off cumulus cells, and transferred into preincubated IVM medium containing 100 nM SiR-tubulin (Cytoskeleton Inc.) and 0.5% Blue Nuclear Stain (Invitrogen, Thermo Fisher Scientific, Saint-Laurent, Quebec, Canada) for 30 minutes. Live images of fluorescence signals with phase contrast were captured under a Zeiss spinning disk Axio Observer Z1 confocal microscope using EC Plan-Neofluar ×10/0.30 WD = 5.2 every 10 minutes for up to 4 hours at the Molecular Imaging Facility of RI-MUHC.

### Statistics.

Statistical analysis was performed using either 2-sided Fisher’s exact test or χ^2^ test on the MedCalc (https://www.medcalc.org/calc/fisher.php) or the SISA website (https://www.quantitativeskills.com/sisa/statistics/). *P* values less than 0.05 were considered significant.

### Study approval.

The study of human subjects was approved by McGill University IRB A01-M07-03A. All the animal procedures were approved by the McGill University Animal Care Committee in accordance with the Canadian Council on Animal Care (application 2012-7064).

### Data availability.

Biallelic variants in patients 1690, 1824, 1954, 1802, 1922, and 2136 were submitted to Leiden Open Variation Database (98) under IDs 00454559, 00454561, 00454560, 00454562, 00454564, and 00454558, respectively. Values for all data points in the article and histograms are reported in the Supporting Data Values file.

## Author contributions

MR identified variants in HFM1 and FOXL2, validated/segregated variants in MAJIN and SYCP2 and 4 variants in Supplemental Table 2, and contributed to enrichment analysis. ML validated/segregated 35 variants in Supplemental Table 2, performed reverse transcriptase PCR in supplemental figures, and generated the data in Figures 7 and 8. ZY identified MEIOB, validated/segregated KASH5 and 39 variants in Supplemental Table 2, and prepared supplemental figures. JHM generated Supplemental Figure 23. PK generated Figure 5. EB and JM processed all raw exomes. ZJG’s data contributed to the hypothesis on the parallel positioning of the spindles. MF referred patient 1802 and provided tissue and its genotype. CB referred patient 1824. RH and AS referred patient 1954, provided tissue, and referred 1 patient in Supplemental Table 2. AH referred patient 439. GM, RAF, and MS provided DNA from patients 1690 and 1689 and HM genotypes. EA referred patient 2136, provided slides for HM, and referred 2 patients in Supplemental Table 2. WB referred 32 patients in Supplemental Table 2. PAB, FA, and IT provided DNA from 5 patients in Supplemental Table 2 and tissues analyzed in Supplemental Figures 17–19. IS performed genotyping in Supplemental Figures 9 and 14. OD compiled Excel sheets of exome data. SA provided DNA from 25 patients, 2 in Supplemental Table 2. JQ referred 4 patients in Supplemental Table 2. JVB identified 1 variant in HFM1. LB performed IVM on additional mice. PM referred 4 patients, 1 in Supplemental Table 2. PS referred 4 patients in Supplemental Table 2. SLT referred 2 patients in Supplemental Table 2. TT designed research studies, analyzed data, performed experiments in Figures 6–8, supervised mouse work, and wrote the manuscript. RS designed research studies, identified variants in MAJIN, SYCP2, and KASH5, performed analysis of data in Figure 4, supervised human genetics work, and wrote the manuscript. The method used to assign the authorship order was based on authors’ contribution to the submitted work and time spent on the project.

## Supplementary Material

Supplemental data

Supplemental table 1

Supplemental table 2

Supplemental video 1

Supplemental video 2

Supporting data values

## Version 1 11/15/2024

Electronic publication
