# Supplementary material for: Defects in meiosis I contribute to the genesis of androgenetic hydatidiform moles
Source: J Clin Invest. 2024 Nov 15;134(22):e170669. doi: 10.1172/JCI170669 (PMC11563684; doi:10.1172/JCI170669)
Supplement: Supplemental data [file jci-134-170669-s066.pdf]

Patient 1690

Total number of variants with MAF ≤ 1%	**2696 variants**
Potentially recessive variants: (homozygous & multiple heterozygous)	**319 variants**
Variants in ≤5 in-house controls	**221 variants**
Frameshift/splicing/stop-gain/ nonsynonymous	**92 variants**
Homozygous/multiple heterozygous with CADD score≥10, remove low ranked genes	**24 genes**
Role in reproduction	***FOXL2***

Two other genes were in the same run of homozygosity as FOXL2, but both were not involved in reproduction

Position	Zygosity	Variant	Gene	CADD	Classification	Full gene name
chr1:17944985	multiple het	p.P46L	ARHGEF10L	18.37	Likely benign	Rho guanine nucleotide exchange factor (GEF) 10-like
chr1:18023372	multiple het	p.C816R	ARHGEF10L	13.7	Likely benign	Rho guanine nucleotide exchange factor (GEF) 10-like
chr1:201837847	hom	p.M643V	IPO9	23.4	Likely benign	importin 9
chr2:74763556	hom	p.R174W	LOXL3	29.4	Uncertain significance	lysyl oxidase-like 3
chr2:108471084	multiple het	p.R351X	RGPD4	37	Uncertain significance	RANBP2-like and GRIP domain containing 4
chr2:108476179	multiple het	p.P546S	RGPD4	13.01	Uncertain significance	RANBP2-like and GRIP domain containing 4
chr3:111940029	hom	p.S491N	SLC9C1	14.16	Uncertain significance	Solute Carrier Family 9 Member C1
chr3:138665065	hom	p.F167S	FOXL2	24.5	Uncertain significance	forkhead box L2
chr3:141905352	hom	p.S208F	GK5	29.3	Uncertain significance	glycerol kinase 5 (putative)
chr9:34723858	multiple het	p.R1127X	FAM205A	35	Uncertain significance	Family With Sequence Similarity 205 Member A
chr9:34725914	multiple het	p.S441R	FAM205A	24.4	Uncertain significance	Family With Sequence Similarity 205 Member A
chr12:25260968	hom	p.R492X	LRMP	38	Uncertain significance	lymphoid-restricted membrane protein
chr12:49722810	multiple het	p.R331H	TROAP	32	Uncertain significance	trophinin associated protein (tastin)
chr12:49723656	multiple het	p.R394Q	TROAP	10.11	Likely benign	trophinin associated protein (tastin)
chr12:116452965	hom	p.H375R	MED13L	15.75	Uncertain significance	mediator complex subunit 13-like
chr14:61747193	multiple het	p.P225S	TMEM30B	19.37	Uncertain significance	transmembrane protein 30B
chr14:61747575	multiple het	p.Y97X	TMEM30B	37	Uncertain significance	transmembrane protein 30B
chr16:88721816	multiple het	p.E230X	MVD	36	Uncertain significance	mevalonate (diphospho) decarboxylase
chr16:88722069	multiple het	p.L225V	MVD	12.64	Uncertain significance	mevalonate (diphospho) decarboxylase
chr17:26864371	hom	p.E622K	FOXN1	26.5	Uncertain significance	forkhead box N1
chr17:27047875	hom	p.K59R	RPL23A	22.6	Uncertain significance	ribosomal protein L23a
chr17:27908976	hom	p.V198I	GIT1	12.85	Benign	G protein-coupled receptor kinase interactor 1
chr17:34199457	hom	p.R67Q	CCL5	34	Uncertain significance	chemokine (C-C motif) ligand 5
chr17:37331496	hom	p.R583C	CACNB1	34	Uncertain significance	calcium handling in skeletal muscle
chr17:39502471	hom	p.C372Y	KRT33A	25.4	Uncertain significance	keratin 33A
chr17:42476045	hom	p.K1056Q	GPATCH8	15.98	Uncertain significance	G patch domain containing 8
chr17:42979917	hom	p.H154P	CCDC103	15.16	Uncertain significance	coiled-coil domain containing 103
chr19:44039535	multiple het	p.R145P	ZNF575	32	Uncertain significance	zinc finger protein 575
chr19:44039540	multiple het	p.Y147H	ZNF575	23.6	Uncertain significance	zinc finger protein 575
chr19:56701308	multiple het	p.R459L	ZSCAN5B	23.2	Uncertain significance	Zinc Finger And SCAN Domain-Containing Protein 5B
chr19:56701355	multiple het	p.Y443X	ZSCAN5B	28.7	Uncertain significance	Zinc Finger And SCAN Domain-Containing Protein 5B
chr19:56701387	multiple het	p.H433Y	ZSCAN5B	24.4	Uncertain significance	Zinc Finger And SCAN Domain-Containing Protein 5B
chr20:44533615	hom	p.A188V	PLTP	34	Uncertain significance	phospholipid transfer protein

Supplemental Figure 1. Exome filtering for potentially recessive variants in patient 1690 (1st WES). Genes in the homozygous interval on chromosome 3 are indicated by a bracket.

Patient 1690 end WES

Filter	Result
Total number of variants with MAF ≤ 1%	2696 variants
Potentially recessive variants: (homozygous & multiple heterozygous)	319 variants
Variants in ≤5 in-house controls	221 variants
Frameshift/splicing/stop-gain/ nonsynonymous	92 variants
Homozygous/multiple heterozygous with CADD score≥10, remove low ranked genes	24 genes
Role in reproduction	***FOXL2***

Two other genes were in the same run of homozygosity as FOXL2, but both were not involved in reproduction

Position	Zygosity	Variant	Gene	CADD	Classification	Full gene name
chr1:17944985	multiple het	p.P46L	ARHGEF10L	18.37	Likely benign	Rho guanine nucleotide exchange factor (GEF) 10-like
chr1:18023372	multiple het	p.C816R	ARHGEF10L	13.7	Likely benign	Rho guanine nucleotide exchange factor (GEF) 10-like
chr1:201837847	hom	p.M643V	IPO9	23.4	Likely benign	importin 9
chr2:74763556	hom	p.R174W	LOXL3	29.4	Uncertain significance	lysyl oxidase-like 3
chr2:108471084	multiple het	p.R351X	RGPD4	37	Uncertain significance	RANBP2-like and GRIP domain containing 4
chr2:108476179	multiple het	p.P546S	RGPD4	13.01	Uncertain significance	RANBP2-like and GRIP domain containing 4
chr3:111940029	hom	p.S491N	SLC9C1	14.16	Uncertain significance	Solute Carrier Family 9 Member C1
chr3:138665065	hom	p.F167S	FOXL2	24.5	Uncertain significance	forkhead box L2
chr3:141905352	hom	p.S208F	GK5	29.3	Uncertain significance	glycerol kinase 5 (putative)
chr9:34723858	multiple het	p.R1127X	FAM205A	35	Uncertain significance	Family With Sequence Similarity 205 Member A
chr9:34725914	multiple het	p.S441R	FAM205A	24.4	Uncertain significance	Family With Sequence Similarity 205 Member A
chr12:25260968	hom	p.R492X	LRMP	38	Uncertain significance	lymphoid-restricted membrane protein
chr12:49722810	multiple het	p.R331H	TROAP	32	Uncertain significance	trophinin associated protein (tastin)
chr12:49723656	multiple het	p.R394Q	TROAP	10.11	Likely benign	trophinin associated protein (tastin)
chr12:116452965	hom	p.H375R	MED13L	15.75	Uncertain significance	mediator complex subunit 13-like
chr14:61747193	multiple het	p.P225S	TMEM30B	19.37	Uncertain significance	transmembrane protein 30B
chr14:61747575	multiple het	p.Y97X	TMEM30B	37	Uncertain significance	transmembrane protein 30B
chr16:88721816	multiple het	p.E230X	MVD	36	Uncertain significance	mevalonate (diphospho) decarboxylase
chr16:88722069	multiple het	p.L225V	MVD	12.64	Uncertain significance	mevalonate (diphospho) decarboxylase
chr17:26864371	hom	p.E622K	FOXN1	26.5	Uncertain significance	forkhead box N1
chr17:27047875	hom	p.K59R	RPL23A	22.6	Uncertain significance	ribosomal protein L23a
chr17:27908976	hom	p.V198I	GIT1	12.85	Benign	G protein-coupled receptor kinase interactor 1
chr17:34199457	hom	p.R67Q	CCL5	34	Uncertain significance	chemokine (C-C motif) ligand 5
chr17:37331496	hom	p.R583C	CACNB1	34	Uncertain significance	calcium handling in skeletal muscle
chr17:39502471	hom	p.C372Y	KRT33A	25.4	Uncertain significance	keratin 33A
chr17:42476045	hom	p.K1056Q	GPATCH8	15.98	Uncertain significance	G patch domain containing 8
chr17:42979917	hom	p.H154P	CCDC103	15.16	Uncertain significance	coiled-coil domain containing 103
chr19:44039535	multiple het	p.R145P	ZNF575	32	Uncertain significance	zinc finger protein 575
chr19:44039540	multiple het	p.Y147H	ZNF575	23.6	Uncertain significance	zinc finger protein 575
chr19:56701308	multiple het	p.R459L	ZSCAN5B	23.2	Uncertain significance	Zinc Finger And SCAN Domain-Containing Protein 5B
chr19:56701355	multiple het	p.Y443X	ZSCAN5B	28.7	Uncertain significance	Zinc Finger And SCAN Domain-Containing Protein 5B
chr19:56701387	multiple het	p.H433Y	ZSCAN5B	24.4	Uncertain significance	Zinc Finger And SCAN Domain-Containing Protein 5B
chr20:44533615	hom	p.A188V	PLTP	34	Uncertain significance	phospholipid transfer protein

Supplemental Figure 2. Exome filtering for potentially recessive variants in patient 1690 (2nd WES). Genes in the homozygous interval on chromosome 3 are indicated by a bracket.

Patient 1824

Filter	Result
Total number of variants with MAF ≤ 1%	**844 variants**
Potentially recessive variants: (homozygous & multiple heterozygous)	**66 variants**
Variants in ≤5 in-house controls	**35 variants**
Frameshift/splicing/stop-gain/ nonsynonymous	**24 variants**
Homozygous/multiple heterozygous CADD score>10	**7 genes**
Role in reproduction	***MAJIN***

Position	Zygosity	Varian	Gene	CADD	Classification	Full gene name
chr1:223989848	hom	p.M399V	*TP53BP2*	10.11	VUS	tumor protein p53 binding protein, 2
chr2:234372897	multiple het	p.L1048F	*DGKD*	25.6	VUS	diacylglycerol kinase, delta 130kDa
chr2:234375851	multiple het	splicing	*DGKD*	26.2	Likely Pathogenic	diacylglycerol kinase, delta 130kDa
chr11:61646028	hom	p.V235M	*FADS3*	28.4	VUS	fatty acid desaturase 3
chr11:64717214	hom	splicing	*MAJIN*	26.4	Likely Pathogenic	Membrane Anchored Junction Protein
chr13:79233230	hom	p.T9I	*RNF219*	28.6	VUS	ORC Ubiquitin ligase
chr15:68937586	hom	p.P30A	*CORO2B*	17.69	VUS	coronin, actin binding protein, 2B
chr20:860454	hom	p.T330S	*ANGPT4*	25	VUS	angiopoietin 4

Only 2 genes were present in the 48.3-Mb run of homozygosity on chromosome 11, of which one had an established role in mice in female infertility.

Supplemental Figure 3. Exome filtering for potentially recessive variants in patient 1824. Genes in the homozygous interval on chromosome 11 are indicated by a bracket.

Patient 439

Filtering step	Result
Total number of variants with MAF ≤ 1%	932 variants
Potentially recessive variants: (homozygous & multiple heterozygous)	165 variants
Variants in ≤5 in-house controls	122 variants
Frameshift/splicing/stop-gain/ nonsynonymous	93 variants
Homozygous/multiple heterozygous Polyphen score ≥0.5 or blank, & excluding low ranking genes	11 genes
Role in reproduction	*KASH5*

Position	Homozygosity	Protein Change	Gene	Polyphen2 score	Gene Description
chr2:108460090	hom	p.R229Q	RGPD4	Blank	RANBP2-like and GRIP domain containing 4
chr2:178494176	possibly hom	p.S476delinsSP	PDE11A	Blank	phosphodiesterase 11A
chr2:233712227	hom	p.P1211delinsQP	GIGYF2	Blank	trinucleotide repeat containing 15
chr3:118865140	multiple het	p.Q35R	C3orf30	0.929	chromosome 3 open reading frame 30
chr3:118865615	multiple het	p.Q193H	C3orf30	0.683	chromosome 3 open reading frame 30
chr6:7373628	multiple het	p.R339Q	CAGE1	Blank	cancer antigen 1
chr6:7374210	multiple het	p.R145Q	CAGE1	Blank	cancer antigen 1
chr7:72733018	multiple het	p.R177C	TRIM50	0.994	tripartite motif-containing 50// tripartite motif-containing 50A
chr7:72738583	multiple het	p.N68I	TRIM50	0.999	tripartite motif-containing 50// tripartite motif-containing 50A
chr8:125989023	multiple het	p.N171K	ZNF572	0.951	zinc finger protein 572
chr8:125989758	multiple het	p.E416D	ZNF572	0.62	zinc finger protein 572
chr15:22742660	possibly hom	p.E349K	GOLGA6L1	Blank	
chr15:22742661	possibly hom	p.E349G	GOLGA6L1	Blank	
chr16:11220018	multiple het	p.R868C	CLEC16A	Blank	C-type lectin domain family 16, member A
chr16:11272435	multiple het	p.S1017N	CLEC16A	Blank	C-type lectin domain family 16, member A
chr19:49920633	multiple het	p.R519X	CCDC155	Blank	KASH Domain-Containing Protein 5
chr19:49920682	multiple het	p.L535Q	CCDC155	Blank	KASH Domain-Containing Protein 5

13 variants in other genes were also selected under less stringent criteria, validated, segregated, and the results allowed their exclusions (either not confirmed in the patient or not segregating with the phenotype).

Supplemental Figure 4. Exome filtering for potentially recessive variants in patient 439.

Patient 1954

Filter	Result
Total number of variants with MAF ≤ 1%	**1643 variants**
Potentially recessive variants: (homozygous & multiple heterozygous)	**239 variants**
Variants in ≤5 in-house controls	**134 variants**
Frameshift/splicing/stop-gain/ nonsynonymous	**83 variants**
Homozygous/multiple heterozygous CADD score>10	**10 genes**
Role in reproduction	***SYCP2***

Position	Zygosity	Variant	Gene	CADD	Classification	Full gene name
chr1:1564775	possibly hom	p.A848T	*MIB2*	10.91	VUS	mindbomb homolog 2 (Drosophila)
chr1:109444428	multiple het	p.A272S	*GPSM2*	25.5	VUS	G-protein signaling modulator 2 (AGS3-like, C. elegans)
chr1:109472390	multiple het	p.D628V	*GPSM2*	29.4	VUS	G-protein signaling modulator 2 (AGS3-like, C. elegans)
chr2:64323264	hom	p.K229E	*PELI1*	16.56	VUS	pellino homolog 1 (Drosophila)
chr6:133846250	hom	p.N558K	*EYA4*	17.78	VUS	eyes absent homolog 4 (Drosophila)
chr9:90321137	hom	p.V1051M	*DAPK1*	23.4	VUS	death-associated protein kinase 1
chr14:70418999	multiple het	p.V82M	*SMOC1*	23.2	VUS	SPARC related modular calcium binding 1
chr14:70478230	multiple het	p.A296T	*SMOC1*	23.6	VUS	SPARC related modular calcium binding 1
chr15:34825151	hom	p.S61A	*GOLGA8B*	15.04	VUS	golgi autoantigen, golgin subfamily a, 8B
chr16:21848618	possibly hom	p.D364Y	*NPIPB4*	13.98	VUS	Nuclear Pore Complex-Interacting Protein Family Member B4
chr19:53269933	multiple het	p.K359R	*ZNF600*	17.48	LB	zinc finger protein 600
chr19:53270257	multiple het	p.C251Y	*ZNF600*	24	VUS	zinc finger protein 600
chr20:58457222	hom	splicing	*SYCP2*	22.5	Pathogenic	synaptonemal complex protein 2

Only one gene in the 19.7-Mb run of homozygosity on chromosome 20

Supplemental Figure 5. Exome filtering for potentially recessive variants in patient 1954.

Patient 1802

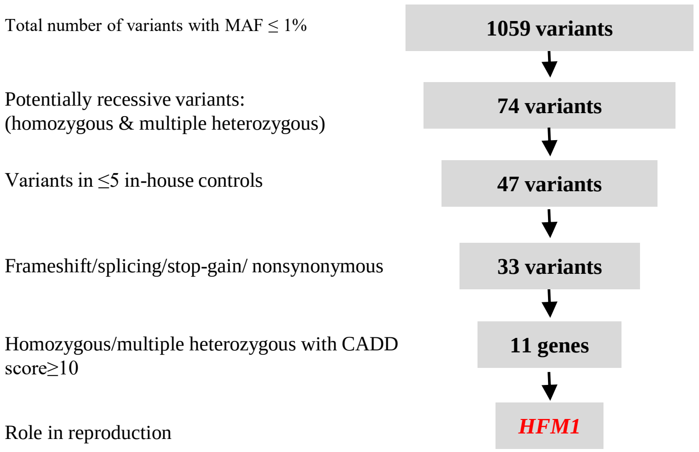

Position	Zygosity	Variant	Gene	CADD	Classification	Full gene name
chr1:89473514	hom	p.E427Q	GBP3	20.7	VUS	guanylate binding protein 3// spastic paraplegia 3A (autosomal dominant)
chr1:89630543	hom	splicing	GBP7	15.8	Benign	guanylate binding protein 7
chr1:91781387	hom	p.Y1042fs	HFM1	0	Pathogenic	HFM1, ATP-dependent DNA helicase homolog (S. cerevisiae)
chr1:93677666	hom	p.L448P	CCDC18	28	VUS	coiled-coil domain containing 18
chr1:110299757	hom	p.A301T	EPS8L3	16.7	VUS	EPS8-like 3
chr2:159481864	multiple het	p.D360Y	PKP4	25.9	VUS	plakophilin 4
chr2:159481865	multiple het	p.D360G	PKP4	19.9	VUS	plakophilin 4
chr3:182735094	hom	p.A553V	MCCC1	23.5	VUS	methylcrotonoyl-Coenzyme A carboxylase 1 (alpha)
chr5:176813232	hom	p.91_97del	SLC34A1	0	Likely benign	solute carrier family 34 (sodium phosphate), member 1
chr11:47312310	hom	p.G1039R	MADD	23.8	VUS	electron-transfer-flavoprotein, alpha polypeptide (glutaric aciduria II)
chr14:39560724	multiple het	p.Y187C	SEC23A	25.1	Likely pathogenic	Sec23 homolog A (S. cerevisiae)
chr14:39565257	multiple het	p.N22K	SEC23A	24.8	VUS	Sec23 homolog A (S. cerevisiae)
chr15:40398284	multiple het	p.E2Q	BMF	24.8	VUS	Bcl2 modifying factor
chr15:40398285	multiple het	p.M1I	BMF	27.3	VUS	Bcl2 modifying factor

Only one pathogenic variant in one gene was present in the 25-Mb homozygous region on chromosome 1.

Supplemental Figure 6. Exome filtering for potentially recessive variants in patient 1802. Genes in the homozygous interval on chromosome 1 are indicated by a bracket.

Patient 1922

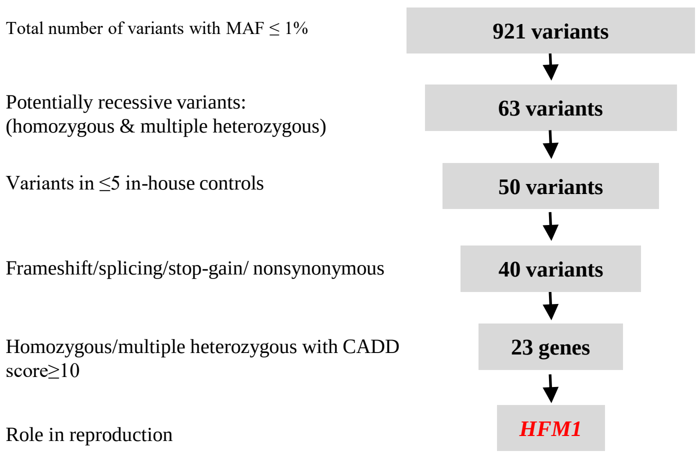

Position	Zygosity	Variant	Gene	CADD	Classification	Full gene name
chr1:16069085	multiple het	p.L11I	TMEM82	20.8	VUS	transmembrane protein 82
chr1:16069570	multiple het	p.E73K	TMEM82	23.7	VUS	transmembrane protein 82
chr1:91781387	multiple het	p.Y1042fs	HFM1	33	LP	HFM1, ATP-dependent DNA helicase homolog (S. cerevisiae)
chr1:91844038	multiple het		HFM1	25.7	LP	HFM1, ATP-dependent DNA helicase homolog (S. cerevisiae)
chr2:179438501	multiple het	p.L15055F	TTN	17.65	VUS	titin
chr2:179654748	multiple het	p.G586D	TTN	18.85	LB	titin
chr4:74352728	multiple het	p.P176L	AFM	29.8	VUS	afamin
chr4:74353539	multiple het		AFM	22.8	VUS	afamin
chr4:90816531	multiple het	p.S137P	MMRN1	22.9	VUS	multimerin 1
chr4:90856311	multiple het	p.Q494X	MMRN1	35	VUS	multimerin 1
chr6:37614063	multiple het	p.R712H	MDGA1	23.5	LB	MAM domain containing glycosylphosphatidylinositol anchor 1
chr6:37623667	multiple het	p.D130H	MDGA1	25.4	VUS	MAM domain containing glycosylphosphatidylinositol anchor 1
chr6:85446768	multiple het	p.M487V	TBX18	21	VUS	T-box 18
chr10:11527059	multiple het	p.T296I	USP6NL	21	VUS	USP6 N-terminal like
chr10:17110649	multiple het	p.915_915del	CUBN	18.88	VUS	cubilin (intrinsic factor-cobalamin receptor)
chr16:21065816	multiple het	p.V1276I	DNAH3	26.4	VUS	dynein, axonemal, heavy chain 3// dynein, axonemal, heavy polypeptide 3
chr16:21123104	multiple het	p.R588W	DNAH3	31	VUS	dynein, axonemal, heavy chain 3// dynein, axonemal, heavy polypeptide 3
chr17:39777046	hom	p.V349E	KRT17	28.2	VUS	keratin 17
chr17:42397440	hom	p.M314V	SLC25A39	26.1	VUS	solute carrier family 25, member 39
chr17:72932029	multiple het	p.A5T	OTOP3	11.51	VUS	otopetrin 3
chr17:72932030	multiple het	p.A5V	OTOP3	13.26	VUS	otopetrin 3
chr21:43522324	hom	p.T412M	UMODL1	25	VUS	uromodulin-like 1
chr21:44179139	hom	p.D64N	PDE9A	26.3	VUS	phosphodiesterase 9A

Supplemental Figure 7. Exome filtering for potentially recessive variants in patient 1922, the maternal aunt of patient 1802.

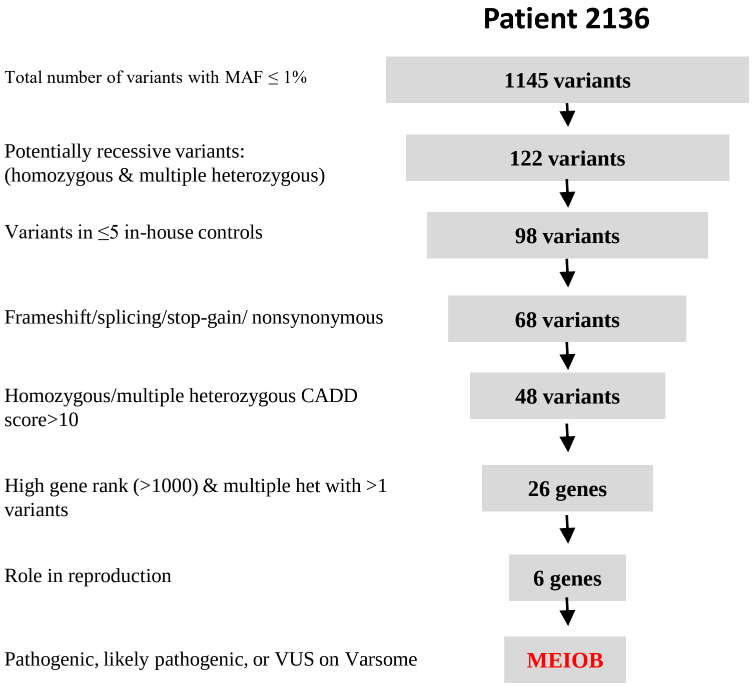

Position	Zygosity	Variant	Gene	CADD score	Classification	Full gene name
chr1:27224070	multiple het	p.R200W	GPATCH3	27.3	Likely Benign	G patch domain containing 3
chr1:27226830	multiple het	p.Q35R	GPATCH3	27.6	Likely Benign	G patch domain containing 3
chr9:34489400	hom	p.D114G	DNAI1	22.8	Likely Benign	Dynein axonemal intermediate chain 1
chr9:35075528	hom	p.H456L	FANCG	24	Likely Benign	Fanconi anemia complementation group G
chr16:1894929	hom	p.R272X	MEIOB	37	Pathogenic	Meiosis specific with OB-fold
chr18:8784510	hom	p.T467M	MTCL1	23.9	Likely Benign	Microtubule crosslinking factor 1
chr19:45867799	hom	p.H177Y	ERCC2	10.02	Benign	ERCC excision repair 2, TFIIH core complex helicase subunit

Supplemental Figure 8. Exome filtering for recessive variants in patient 2136

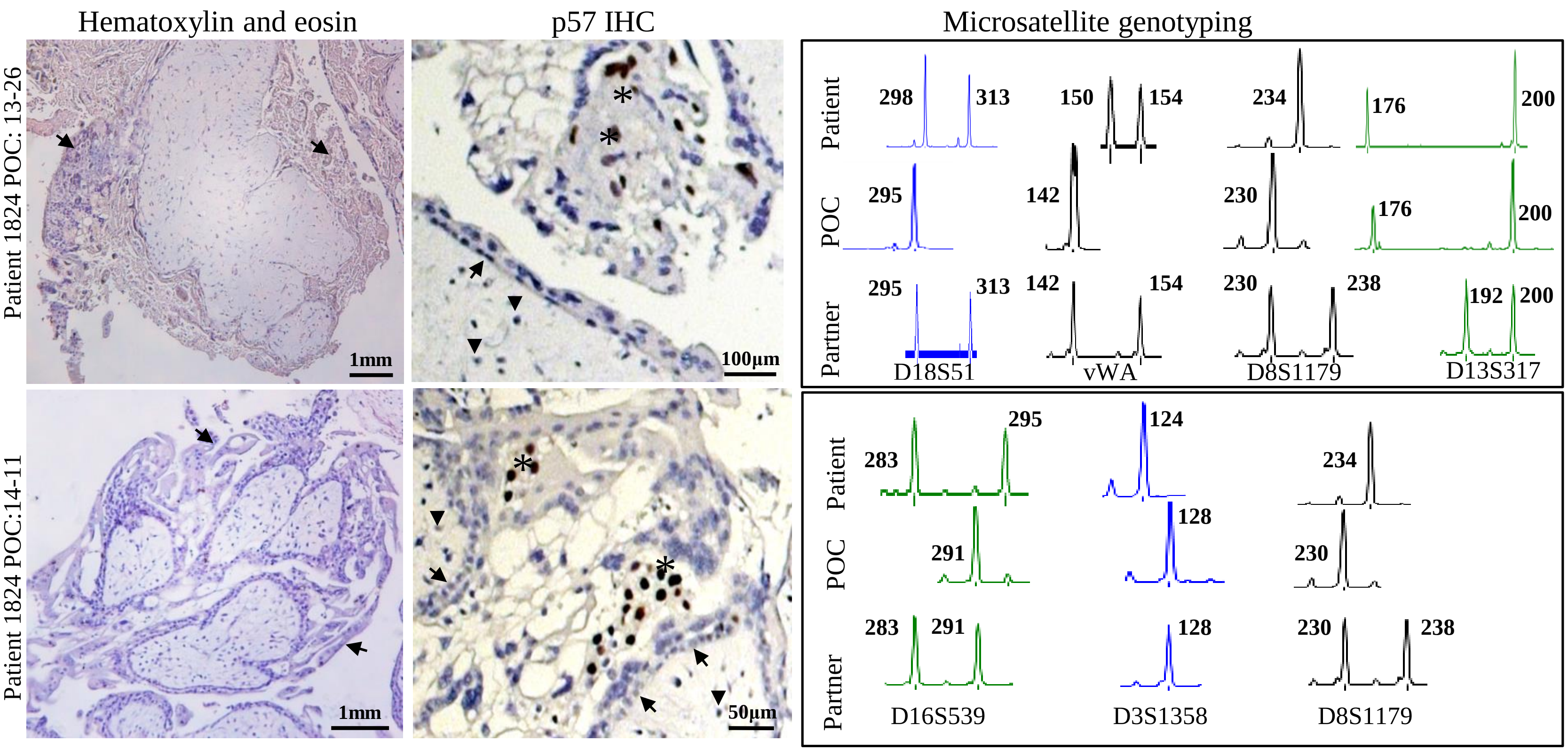

Supplemental Figure 9. Characterization of two HM tissues from patient 1824 with biallelic likely pathogenic variant in *MAJIN*. Left panel shows H&E staining demonstrating circumferential trophoblastic proliferation in the two products of conception (POCs) (arrows). Middle panel shows p57KIP2 IHC showing negative staining in the cytotrophoblast (arrows) in the two POCs of the patient while the internal control, the nuclei of the extravillous trophoblast cells are positive (asterisks) in the two POCs. Right panel shows the genotypes of the two CHM of patient 1824 demonstrating their androgenetic monospermic. In the two POCs, 3 microsatellite markers demonstrate the presence of only one paternal allele in the molar genome at each marker. Note the presence of the maternal chromosome 13 (allele 176bp) in POC 13-26.

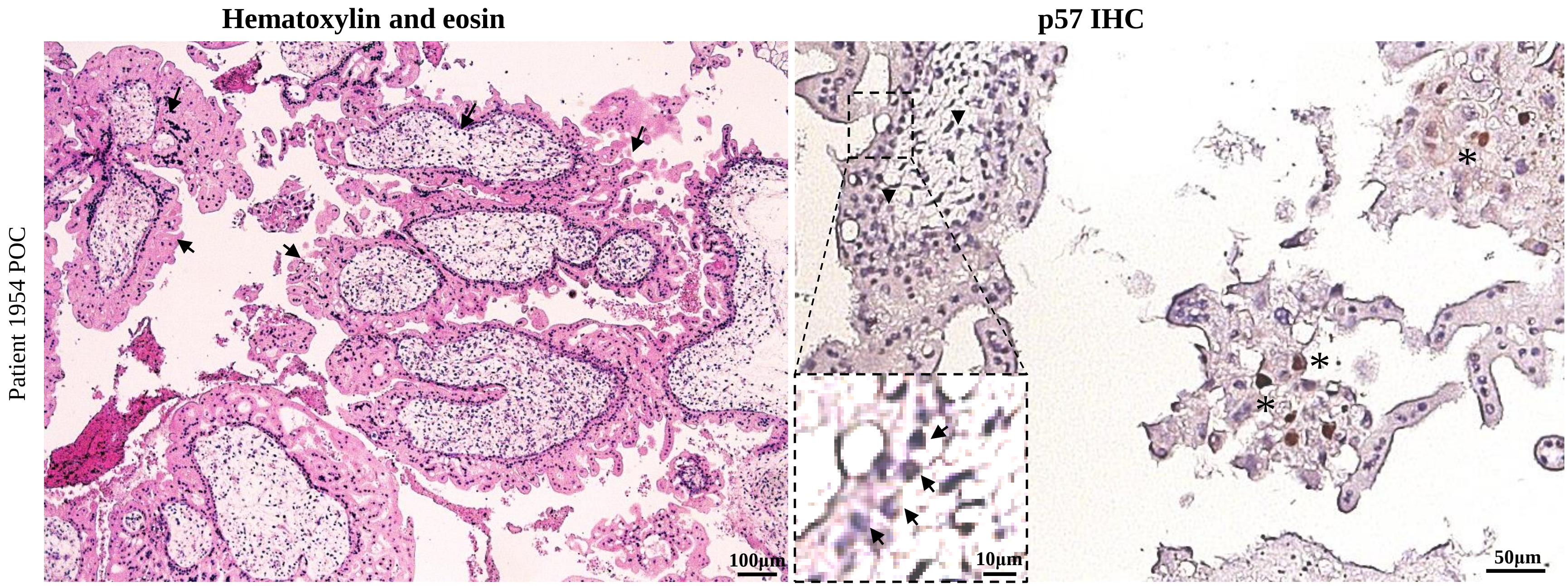

Supplemental Figure 10. Morphological evaluation of one HM from patient 1954 with a biallelic *SYCP2* pathogenic variant. Left panel shows H&E staining demonstrating trophoblastic proliferation in the product of conception (POC) (arrows). Right panel shows p57KIP2 IHC showing negative staining in the cytotrophoblast (arrows in the inset), while the internal control, the nuclei of the extravillous trophoblast cells are positive (asterisks).

A

Hematoxylin and eosin

Patient 1802 POC

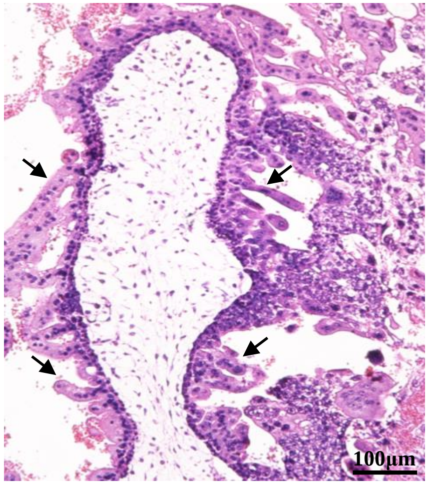

B

Genotyping data of the molar tissue of the affected sister 1921 and the two parents at eight polymorphic microsatellite markers

Marker	Chr.	Partner	POC	Patient 1802
13C	13	191, 194	194	190, 202
13D	13	248, 264	248	259, 275
18A	18	349, 364	349	356, 292
21A	21	290, 298	298	282, 294
21D	21	131	131	119, 130
21E	21	240, 250	240	257
X1	X	187	187	196
X3	X	298	298	290, 294

Chr stands for chromosome and POC for product of conception. Allele sizes are in bp.

Supplemental Figure 11. Morphology of one HM from patient 1921 (the affected sister of patient 1802) with a bi-allelic pathogenic variant in *HFM1*. (**A**) The photo shows a chorionic villous with excessive circumferential trophoblastic proliferation (arrows) typical of HM. (**B)** Genotype data of the same product of conception (POC) shown in A, along with parental DNA, revealed one single paternal allele at each marker demonstrating the androgenetic monospermic genome of the mole. Under marker, the first two digits designate the chromosome number, example, 13C is a marker from chromosome 13, 13D is another marker from the same chromosome, and so on.

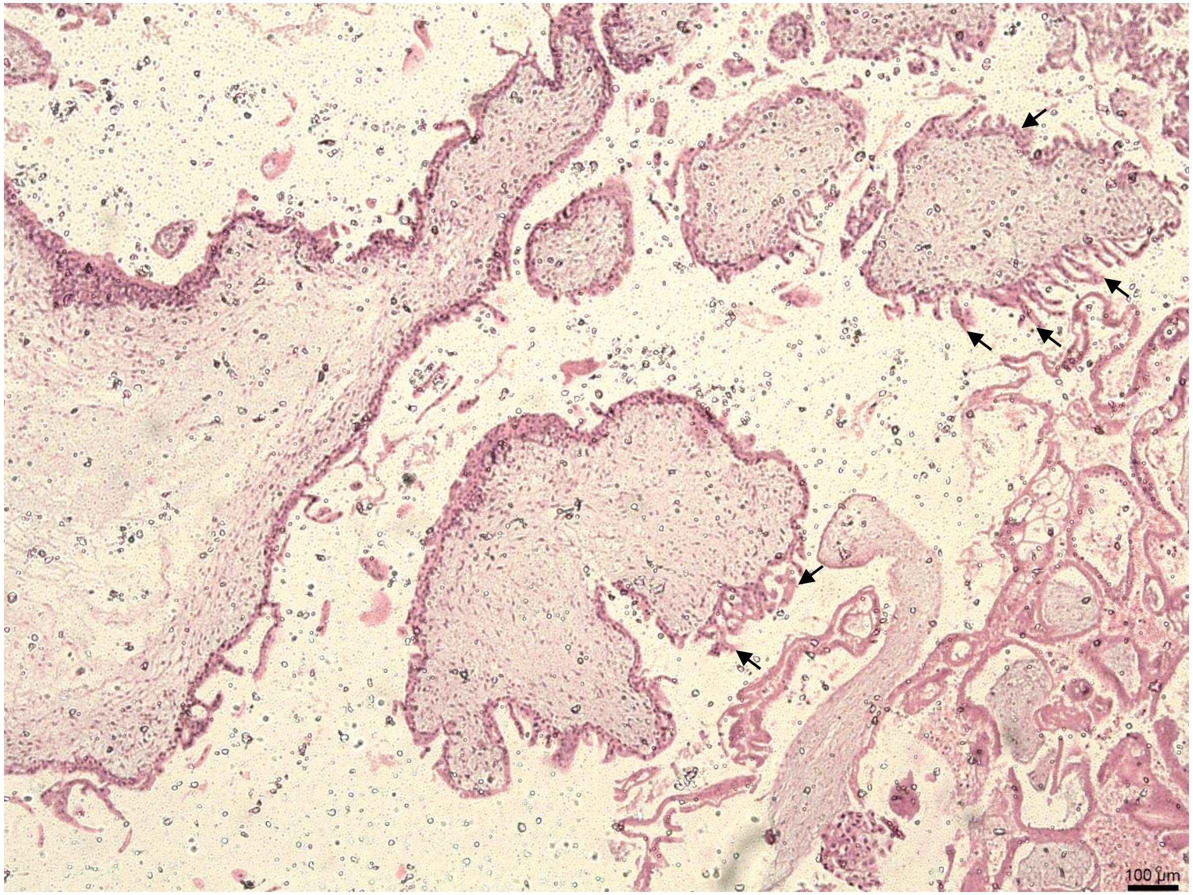

Supplemental Figure 12. Morphology of the HM from patient 2136 with a biallelic *MEIOB* pathogenic variant. H&E staining demonstrating excessive circumferential trophoblastic proliferation projections from the chorionic villi (arrows).

	Alleles			
Locus	1690	CHM3	CHM4	CHM5
D8S1179	15	13	13	13
D21S11	30 - 31.2	31.2	31.2	29
D7S820	10 - 11	8	8	8
CSF1PO	10 - 11	12	12	12
D3S1358	14 - 17	14	15	15
THO1	9 - 9.3	7	7	7
D13S317	9 - 11	13	14	13
D16S539	12 - 13	11	10	11
D2S1338	18 - 20	18	18	18
D19S433	12 - 15	16	15	15
vWA	15 - 19	16	16	16
TPOX	11 - 12	8	8	12
D18S51	13 - 15	15	15	15
AMEL	X	X	X	X
D5S818	10 - 13	13	13	13
FGA	23 - 25	22	22	25

Supplemental Figure 13. Multiplex genotype analysis of three CHM from patient 1690 with a biallelic predicted deleterious variant in *FOXL2*. Genotype data of the three molar conceptions, along with patient DNA, revealed single non-maternal alleles at several markers demonstrating the androgenetic monospermic genome of the mole

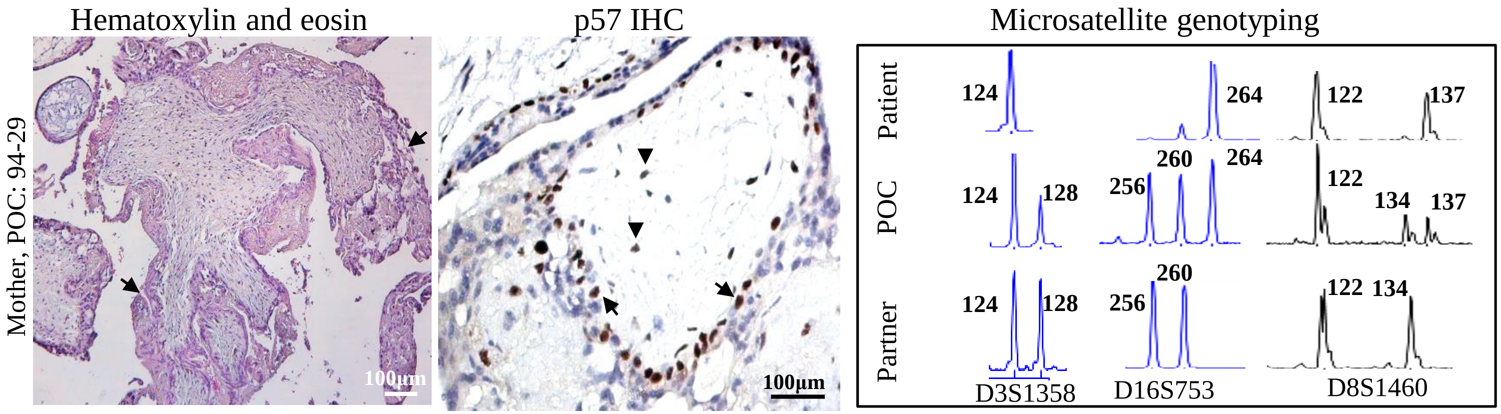

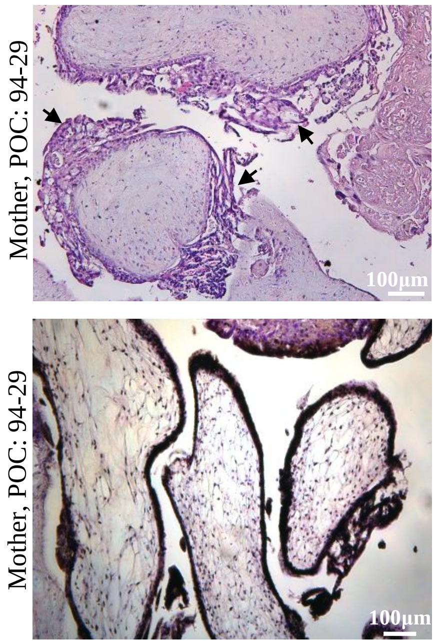

Supplemental Figure 14. Characterization of one HM tissue from the mother of patient 1824 (ID 1825) who is a carrier of a monoallelic variant in *MAJIN*. Left panel shows three H&E staining photos showing chorionic villi with (top two) and without (bottom) circumferential trophoblastic proliferation (arrows). Middle panel shows p57KIP2 IHC showing positive staining in the cytotrophoblast (arrows). Right panel shows the genotype the HM demonstrating its triploid dispermic origin and therefore its diagnosis as PHM.

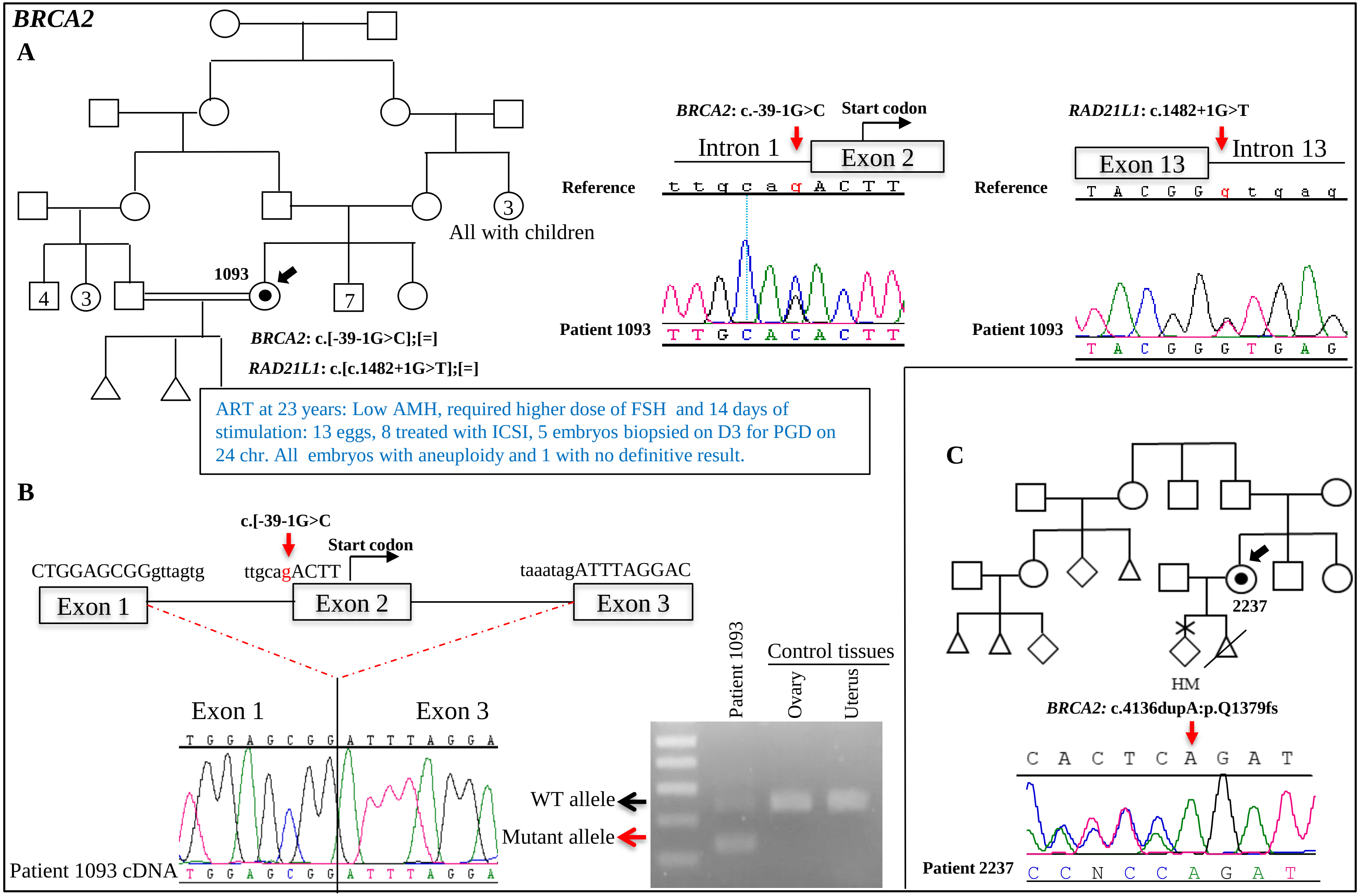

Supplemental Figure 15. A. Pedigree structure of the family of patient 1093 showing the chromatograms of her two variants in *BRCA2* and *RAD51L* along her reproductive history and additional medical information. B. cDNA analysis demonstrated the skipping of exon 2 containing the start codon from the transcripts. C. Pedigree structure of patient 2237 and the chromatogram showing her variant in *BRCA2*.

BRCA2

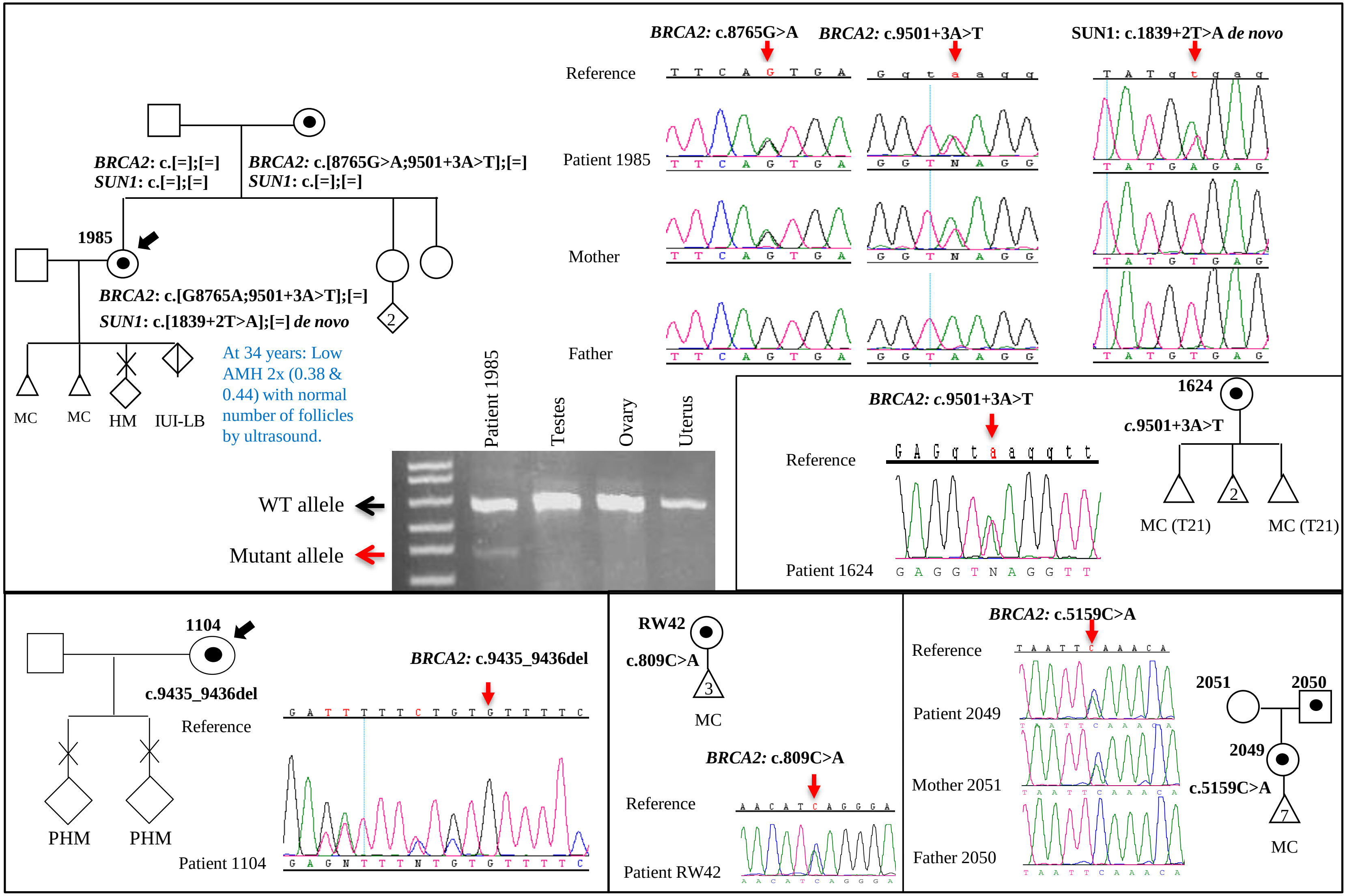

Supplemental Figure 16. Reproductive history and pedigree structure of the family of patient 1985 showing the segregation of her two variants in *BRCA2* (a missense and an extended splice site, c.9501+3A>T in cis) and the *do novo* occurrence of a variant in *SUN1* along with the chromatograms and additional medical information. The identity of the parents of the patient was confirmed using multiplex microsatellite genotyping. cDNA analysis of the splice variant in *BRCA2*, c.9501+3A>T, demonstrating the skipping of exon 25 in a fraction of transcripts from EBV lymphoblastoid cell line from the patient. Chromatograms of four other patients, 1624, 1104, RW42, and 2049, showing their variants and reproductive histories.

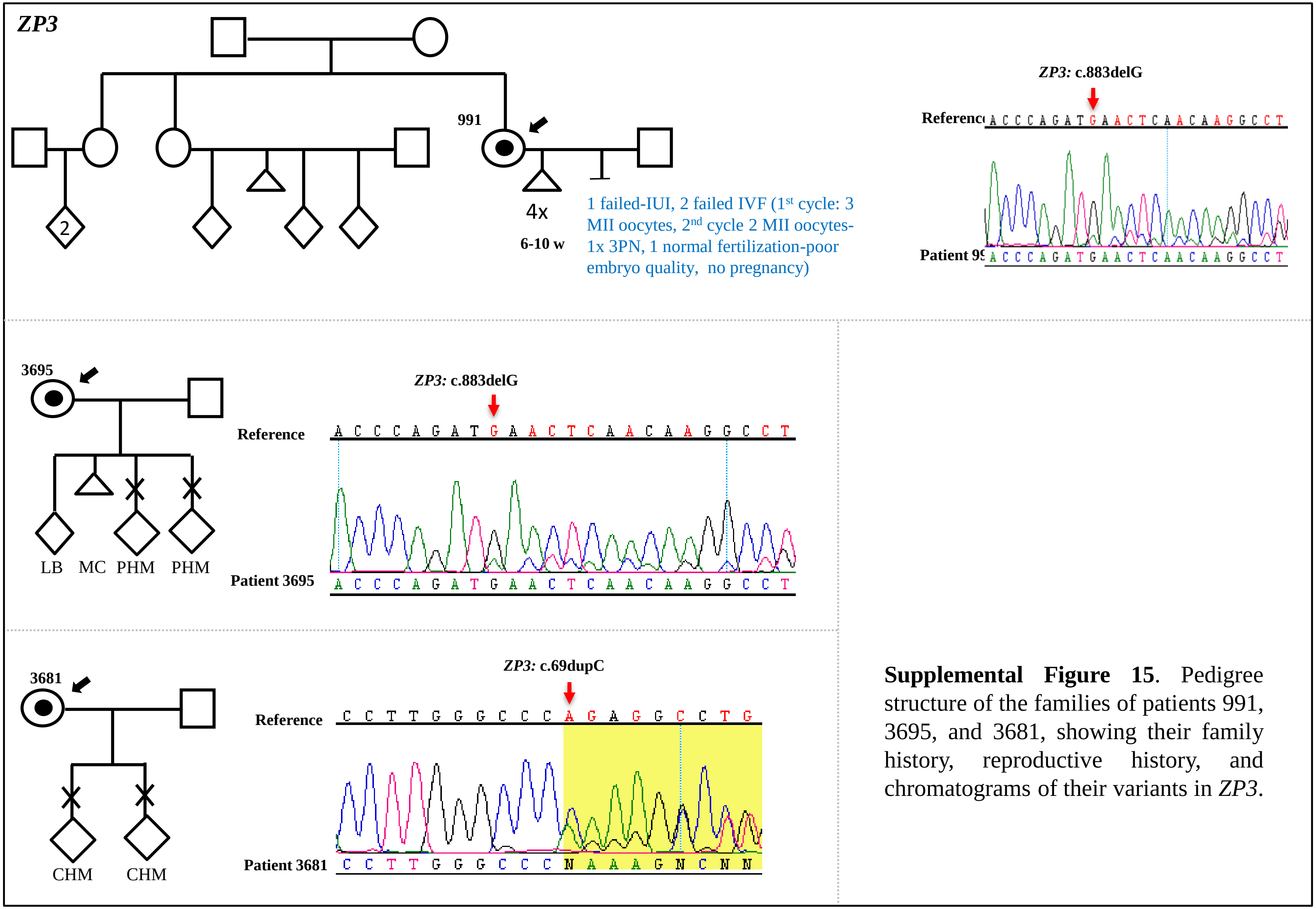

Supplemental Figure 15. Pedigree structure of the families of patients 991, 3695, and 3681, showing their family history, reproductive history, and chromatograms of their variants in *ZP3*.

Supplemental Figure 17

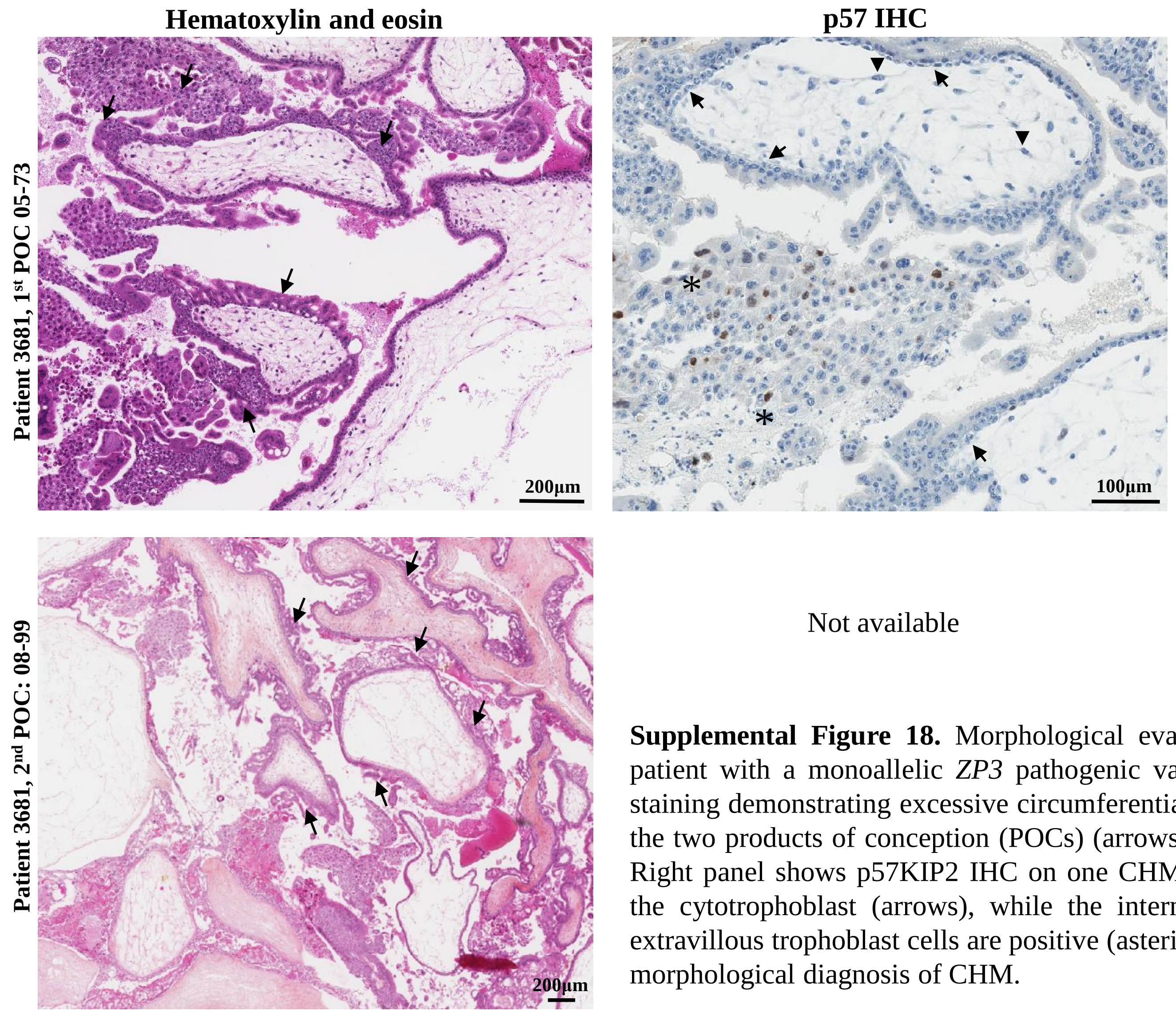

Supplemental Figure 18. Morphological evaluation of two HM from the patient with a monoallelic *ZP3* pathogenic variant. Left panel shows H&E staining demonstrating excessive circumferential trophoblastic proliferation in the two products of conception (POCs) (arrows) and their diagnosis as CHM. Right panel shows p57KIP2 IHC on one CHM showing negative staining in the cytotrophoblast (arrows), while the internal control, the nuclei of the extravillous trophoblast cells are positive (asterisks). P57 IHC corroborates the morphological diagnosis of CHM.

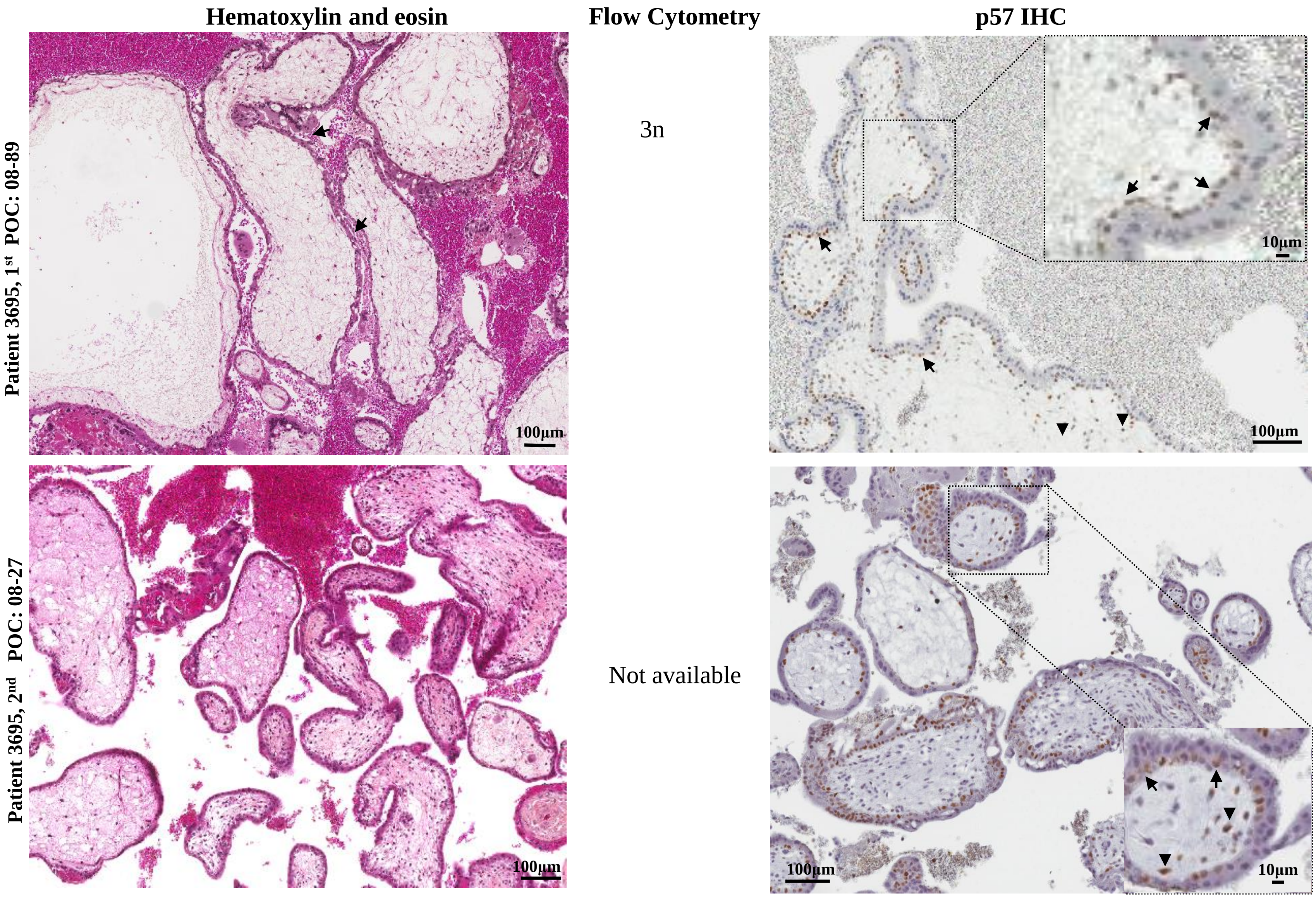

Supplemental Figure 19. Morphological evaluation of two HMs from patient 3695 with a monoallelic *ZP3* VUS P variant. Left panel shows H&E staining demonstrating trophoblastic proliferation in the products of conception (POCs) (arrows). Middle panel shows flow cytometry data on one POC. Right panel shows p57KIP2 IHC showing positive staining in the cytotrophoblast (arrows), and in the internal control (asterisks) in the nuclei of the extravillous trophoblast cells.

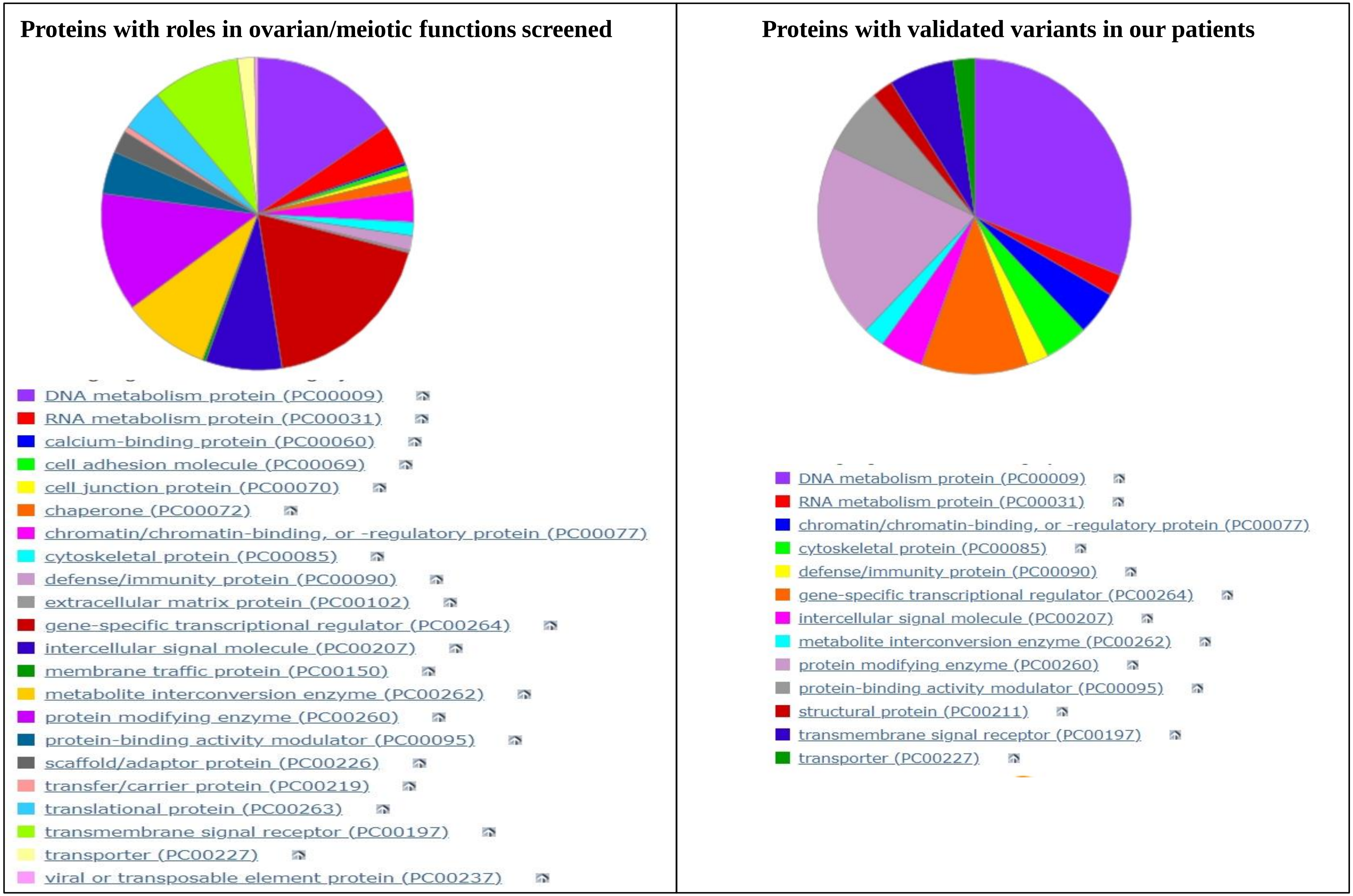

Supplemental Figure 20. Panther classification of the 482 genes screened in our patients and those with monoallelic P/LP variants validated in our patients showing all pathways. These same pies are provided in Figure 4 in the manuscript.

Panther classified ovarian/meiotic genes (n=482) screened for protein truncating variants in our patients	Percentage of genes in class		Panther classification of ovarian/meiotic genes (n=54) whose variants were confirmed by Sanger sequencing in our patients	Percentage of genes in class
Gene-specific transcriptional regulator (PC00264)Intercellular signal molecule (P(	13.1		DNA metabolism protein (PC00009)	24.6
DNA metabolism protein (PC00009)	11		Protein modifying enzyme (PC00260)	15.8
Protein modifying enzyme (PC00260)	8.7		Gene-specific transcriptional regulator (PC00264)Intercellular signal molecule (P(	8.8
Metabolite Interconversion enzyme (PC00262)	6.4		Protein-binding activity modulator (PC00095)	5.3
Transmembrane signal receptor	6.4		Transmembrane signal receptor	5.3
Intercellular signal molecule (PC00207)	5.6		Chromatin/chromatin-binding or regulatory protein (PC00077)	3.5
Protein-binding activity modulator (PC00095)	3.1		Cytoskeletal protein (PC00085)	3.5
Translational protein (PC00263)Transmembrane signal receptor (PC00197)	3.1		Intercellular signal molecule (PC00207)	3.5
RNA metabolism protein (PC00031)	2.9		RNA metabolism protein (PC00031)	1.8
Chromatin/chromatin-binding or regulatory protein (PC00077)	2.3		Defense/immunity protein (PC00090)	1.8
Scaffold/adaptor protein (PC00226)	1.7		Metabolite Interconversion enzyme (PC00262)	1.8
Transporter (PC00227)	1.2		Structural protein (PC00211)	1.8
Chaperon (PC00072)	1			
Cytoskeletal protein (PC00085)	1			
Defense/immunity protein (PC00090)	1			
Cell adhesion molecule (PC00069)	0.4			
Cell junction protein (PC00070)	0.4			
Transfer/carrier protein (PC00219)	0.4			
Calcium-binding protein (PC00060)	0.2			
Extracellular matrix protein (PC00102)	0.2			
Membrane traffic protein (PC00150)	0.2			
Viral or transposable element protein (PC00237)	0.2			
Structural protein (PC00211)	0			

Supplemental Figure 21. Panther Pathway classification of the 482 genes screened in our patients and the number of genes in each class (left) and those with monoallelic P/LP variants validated in our patients (right) along with the statistics. Statistics were performed using the Peason's Chi or Fisher tests as appropriate. P value <0.05 was considered significant.

Panther protein class	Panther classified ovarian/meiotic genes (n=482) screened for protein truncating variants in our patients			Panther classification of ovarian/meiotic genes (n=57) whose variants were confirmed by Sanger sequencing in our patients			Statistical significance
	Percentage of genes in class	N. of gene in class	N. of genes not in this class	Percentage of genes in class	N. of gene in class	N. of genes not in this class	Chi square by Pearson/Fisher test as
Gene-specific transcriptional regulator (PC002	13.1	63	419	8.8	5	52	ns
DNA metabolism protein (PC00009)	11	53	429	24.6	14	43	p=0.0033
Protein modifying enzyme (PC00260)	8.7	42	440	15.8	9	48	ns
Metabolite Interconversion enzyme (PC00262	6.4	31	451	1.8	1	56	ns
Transmembrane signal receptor (PC00197)	6.4	31	451	5.3	3	54	ns
Intercellular signal molecule (PC00207)	5.6	27	455	3.5	2	55	ns
Protein-binding activity modulator (PC00095)	3.1	15	467	5.3	3	54	ns
Translational protein (PC00263)	3.1	15	467				
RNA metabolism protein (PC00031)	2.9	14	468	1.8	1	56	ns
Chromatin/chromatin-binding or regulatory protein (PC00077)	2.3	11	471	3.5	2	55	ns
Scaffold/adaptor protein (PC00226)	1.7	8	474				
Transporter (PC00227)	1.2	6	476	1.8	1	56	ns
Chaperon (PC00072)	1	5	477				
Cytoskeletal protein (PC00085)	1	5	477	3.5	2	55	ns
Defense/immunity protein (PC00090)	1	5	477	1.8	1	56	ns
Cell adhesion molecule (PC00069)	0.4	2	480				
Cell junction protein (PC00070)	0.4	2	480				
Transfer/carrier protein (PC00219)	0.4	2	480				
Calcium-binding protein (PC00060)	0.2	1	481				
Extracellular matrix protein (PC00102)	0.2	1	481				
Membrane traffic protein (PC00150)	0.2	1	481				
Viral or transposable element protein (PC002:	0.2	1	481				
Structural protein (PC00211)	0	0	482	1.8	1	56	

Supplemental Figure 22. Percentages of the different pathways of the 494 screened genes and those that have P/LP variants validated in our patients.

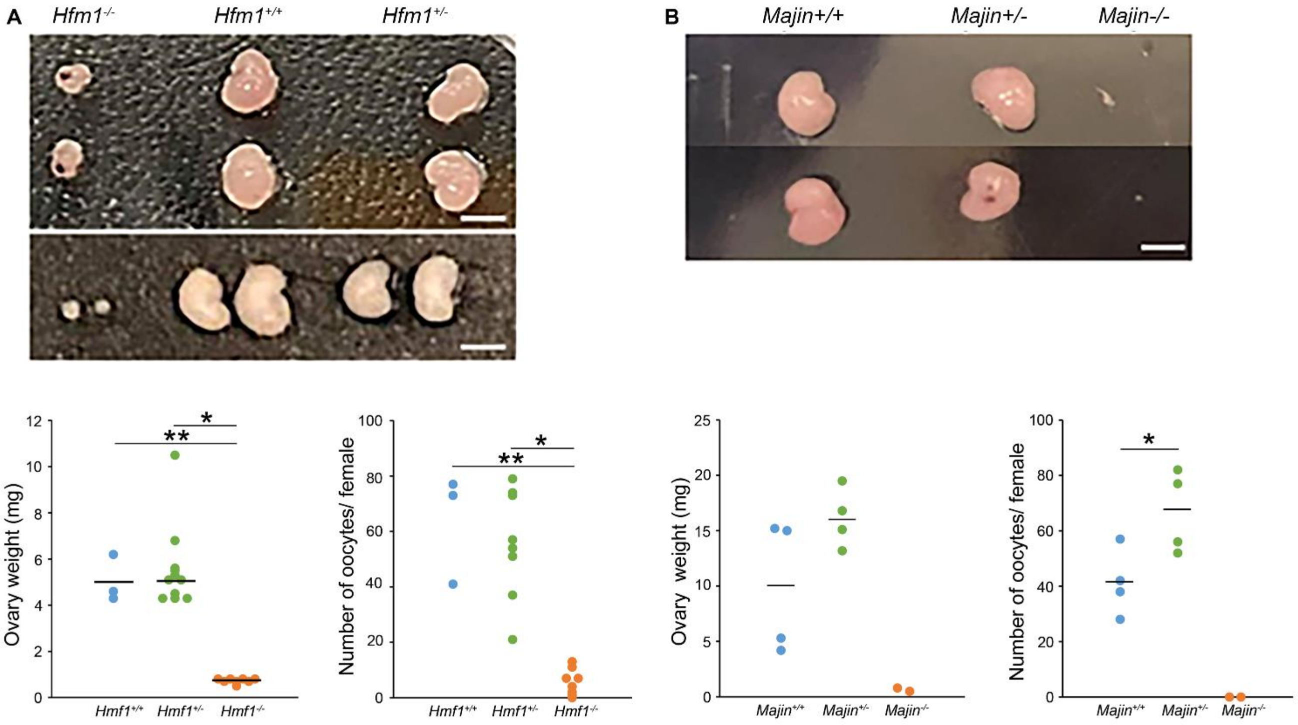

Supplemental Figure 23. A. Gross morphological of ovaries from *Hfm1-/-*, *Hfm1+/-*, and *Hfm1+/+* at 21-24 dpp showing the significantly small ovarian sizes of *Hfm1-/-*. Lower panel shows ovarian weight and number of collected oocytes after superovulation. B. Gross morphological of ovaries from *Majin+/+*, *Majin+/-*, and *Majin-/-* at 21-24 dpp showing the complete loss of the ovaries in *Majin-/-*. Bar 2mm. Lower panel shows ovarian weight and number of collected oocytes after superovulation. *P<0.05 and ** P< 0.001. Statistics were determined using one-way ANOVA or Student's t-test.

Supplemental Table 3. Statistical analysis of alleles frequencies in patients and gnomAD v4.1.0					
**BRCA2**					
	Number of P/LP variants	Number of wild-type alleles	Fisher exact test		
Our patients (n=311)	5	617			
gnomAD (v4.1.0)	1213	1014643			
**p value**			**0.00101792**		
**ZP3**					
	Number of P/LP variants	Number of wild-type alleles	Fisher exact test		
Our patients (n=311)	3	619			
gnomAD (v4.1.0)	301	993960			
**p value**			**0.00098039**		
The two tailed Fisher exact test was performed using the following webiste, https://www.medcalc.org/calc/fisher.php					

Supplemental Table 4. Primary and secondary antibodies used.

To identify meiotic substages

	Host	Source	Catalog No.	Concentration
**Primary antibodies**				
TRA98	rat	AbCam	ac82527	1: 1000
SYCP3	rabbit	Abcam	ac15093	1: 500
CREST	human	Immunovision	HCT-0100	1: 2000
γH2AX	mouse	Millipore	05-636	1: 1000
**Secondary antibodies**				
rat IgG-FITC	goat	Jackson ImmunoResearch	112-545-167	1: 1000
rabbit IgG-rhodamin	goat	PIERCE	31670	1: 1000
human IgG-rho	goat	PIERCE	31656	1: 1000
mouse IgG-AF647	goat	Invitrogen	A-21236	1: 1000

To visualize MLH1 foci

	Host	Source	Catalog No.	Concentration
**Primary antibodies**				
SYCP3	rabbit	Abcam	ac15093	1: 500
CREST	human	Immunovision	HCT-0100	1:1000
MLH1	mouse	BD Pharmigen	550838	1: 50
**Secondary antibodies**				
rabbit IgG-biotin	goat	Invitrogen	31823	1:1000
human IgG-DTAF	goat	PIERCE	31528	1:1000
mouse IgG-FITC	goat	Jackson ImmunoResearch	115-095-003	1:1000
**Streptavidin-rhodamine**		Invitrogen	S6366	1: 1000

To identify oocytes and follicles

	Host	Source	Catalog No.	Concentration
**Primary antibodies**				
FOXL2	goat	Novus	NB100-1277	1:1000
MSY2	rabbit	AbCam	ab3164	1: 500
**Secondary antibodies**				
goat IgG-RRX	donkey	Jackson ImmunoResearch	705-295-003	1:1000
rabbit IgG-AF488	donkey	Invitrogen	A21206	1:1000

To examine oocytes after in vitro maturation

	Host	Source	Catalog No.	Concentration
α-tubulin-AF488	mouse	Invitrogen	#53-4502-82	1: 500
